# A benchmark-driven approach to reconstruct metabolic networks for studying cancer metabolism

**DOI:** 10.1371/journal.pcbi.1006936

**Published:** 2019-04-22

**Authors:** Oveis Jamialahmadi, Sameereh Hashemi-Najafabadi, Ehsan Motamedian, Stefano Romeo, Fatemeh Bagheri

**Affiliations:** 1 Department of Biotechnology, Faculty of Chemical Engineering, Tarbiat Modares University, Tehran, Iran; 2 Department of Biomedical Engineering, Faculty of Chemical Engineering, Tarbiat Modares University, Tehran, Iran; 3 Department of Molecular and Clinical Medicine, University of Gothenburg, Gothenburg, Sweden; 4 Clinical Nutrition Unit, Department of Medical and Surgical Sciences, Magna Graecia University, Catanzaro, Italy; 5 Cardiology Department, Sahlgrenska University Hospital, Gothenburg, Sweden; University of Chicago, UNITED STATES

## Abstract

Genome-scale metabolic modeling has emerged as a promising way to study the metabolic alterations underlying cancer by identifying novel drug targets and biomarkers. To date, several computational methods have been developed to integrate high-throughput data with existing human metabolic reconstructions to generate context-specific cancer metabolic models. Despite a number of studies focusing on benchmarking the context-specific algorithms, no quantitative assessment has been made to compare the predictive performance of these methods. Here, we integrated various and different datasets used in previous works to design a quantitative platform to examine functional and consistency performance of several existing genome-scale cancer modeling approaches. Next, we used the results obtained here to develop a method for the reconstruction of context-specific metabolic models. We then compared the predictive power and consistency of networks generated by our method to other computational approaches investigated here. Our results showed a satisfactory performance of the developed method in most of the benchmarks. This benchmarking platform is of particular use in algorithm selection and assessing the performance of newly developed algorithms. More importantly, it can serve as guidelines for designing and developing new methods focusing on weaknesses and strengths of existing algorithms.

## Author summary

Several attempts have been made to develop computational approaches to integrate high-throughput omics data with generic models of human metabolism. However, no comprehensive and quantitative platform is available to examine the performance of these methods both functionally and structurally. Here, we collected numerous datasets to benchmark some of the context-specific methods used to study the cancer metabolism in order to provide a platform for future algorithm selection, comparison, or algorithm design. Utilizing the performance comparison results, we took a benchmark-driven approach to develop a context-specific reconstruction algorithm based on the advantageous features of algorithms studied here. The promising performance of our method may provide the opportunity for feature algorithm design studies on cancer metabolism.

## Introduction

The advent of next-generation sequencing has shed light on a myriad of mutational events occurring in cancer-related genes. However, deciphering the associated mechanisms underlying the phenotypic alterations cannot be solely deduced from these mutational events due to both extensive heterogeneity of cancer cells and the complexity of biological networks [1, 2]. One emerging way to interpret omics measurements and unravel the complexity of cancer metabolism is to employ genome-scale metabolic models (GEMs) to understand how the cancer metabolism responds to environmental and genetic stresses [3, 4]. General human metabolic models encompass all possible biochemical reactions that are known to occur in different human tissues and cells [5–7]; and hence, this generality in human GEMs implicates their non-specificity to any human tissue or cell. The integration of numerous available cancer high-throughput omics data with global human GEMs provides an invaluable opportunity to study metabolic alterations of cancer cells, and to discover novel drug targets and biomarkers via reconstruction of tissue/cell specific (context-specific) GEMs [2, 8, 9]. To date, many context-specific reconstruction algorithms have been developed for data integration with general GEMS [10–19], and several publications have reviewed the scope of these algorithms from perspectives ranging from mathematical properties to their applicability in the realm of cancer metabolism [1–3, 8, 20]. Nevertheless, none of these publications quantitatively compared the predictive power of these algorithms using common benchmarks and experimental datasets. Recently, a number of studies have undertaken the challenge of designing and introducing methods to benchmark existing context-specific reconstructions [21–24]. Machado and Herrgård [21] comprehensively compared the predictive ability of several methods in terms of internal fluxes, growth and uptake/secretion rates for *Escherichia coli* and yeast. Moreover, they compared the results obtained with those of parsimonious FBA (pFBA) to evaluate the impact of omics integration with the GEM under study. In another study, Pacheco *et al*. [22] introduced a benchmarking method for the quality evaluation of context-specific algorithms consisting of comparison and consistency based methods with a focus on the latter. Very recently, Opdam *et al*. [23] systematically assessed the impact of algorithm assumptions including parameter selection, expression thresholds and metabolic constraints on predictive capability of generated context-specific models for four cancer cell lines. In a similar study, Ferreira *et al* used transcriptomics and proteomics datasets to reconstruct cell-specific GEMs of healthy liver and hepatocellular carcinoma (HCC) cells using four different algorithms. Functional and structural analysis of these models revealed that none of the examined algorithms were ideal based on comparison results [24].

Although these studies paved the way toward systematic evaluation of the developed context-specific algorithms, the guidelines, and extensive comparison based benchmarks for prospective development of new algorithms in the field of cancer metabolism have not been thoroughly investigated.

Here, we have first extracted several experimental datasets, namely cancer essential genes, growth rates, oncogenes and tumor suppressors, drug responses and metabolite uptake/secretion rates from previous studies on context-specific reconstruction algorithms. These datasets represent our comparison based tests for cancer GEMs. We also adapted the consistency based tests from previous benchmarking studies to further illuminate the structural characteristics of generated networks [21, 22]. Through these set of tests, we examined several algorithms in terms of functional and structural properties. Furthermore, we used the results obtained from these benchmarks to identify the bottlenecks of the selected algorithms and to choose the most appropriate ones for use in the realm of metabolic modeling of cancer. Finally, we took a benchmark based approach to develop a context-specific reconstruction algorithm based on incorporation of successful properties of the most accurate algorithms examined in this study. To the best of our knowledge, this is the first time that such a benchmark driven approach is employed to develop an algorithm based on extensive evaluation of its previous ancestors.

## Methods

### General human model setup

The consistent part of Recon 1 (i.e., all blocked reactions, which were unable to carry a non-zero flux under any simulation conditions, were removed) was used as a general input model for generating context-specific models [6, 16]. The biomass function and growth medium (RPMI-1640) for Recon 1 were taken from Folger *et al* [25]. Since the measured metabolite uptake/secretion rates showed the secretion of alanine and glutamate [26, 27], associated exchange reactions were not constrained in input models (as constrained before in Folger *et al* [25]).

To account for the impact of constraining the input model with cell-specific phenotypic data, GEMs were generated using metabolite uptake/secretion rates measured in Jain *et al* [27] study (CORE data), and compared to models generated using above-mentioned general medium. These measurements were converted to usable unit of uptake rate (mmol gDW^-1^hr^-1^) as follows (Eq 1).

$$v_{met,i}^{c}=\frac{4.3\times C_{met,i}}{V^{c}}$$ (1)

where C_met,i_ is the exchange rate of metabolite *i* in the medium (fmol cell^-1^hr^-1^), the coefficient 4.3 is the cell specific volume (mL gDW^-1^) taken from Frame and Hu study [28], V^c^ is the cell volume measured by Dolfi *et al* [29] (fL cell^-1^), and $v_{met,i}^{c}$ is the upper bound of uptake rate of metabolite *i* for the cell line *c* (mmol gDW^-1^hr^-1^). GEMs generated using these cell-specific uptake rates are denoted by the superscript “C” to be distinguishable from the models generated using the general medium. It should be noted that, since some metabolites existing in simulated RPMI-1640 were absent from CORE data, the uptake rates from the general medium were used to fill the missing exchange rates.

### Algorithms setup

All *in silico* simulations were carried out on a 24 core SuperMicro system with 32 GB RAM, using MATLAB R2017b (The MathWorks, Natick, USA) with Gurobi Optimizer 5.5 (Gurobi Optimization, Inc.) as solver. Depending on the algorithm, COBRA [30] or RAVEN toolboxes [31] were employed. For all algorithms, input general human model was constrained with the above-mentioned uptake rates in the medium prior to the reconstruction process.

#### pFBA

The existing implementation in the COBRA toolbox was employed, and L_1_-norm of flux distribution was minimized to reduce the number of optimal flux distributions [32]. The objective function was set to biomass generation through all simulation scenarios.

#### GIMME

The existing implementation of GIMME in COBRA toolbox was slightly modified to account for the direct use of expression values as weights in the objective function, as in the original study [17]. The fraction of objective function and the gene expression threshold were selected based on sensitivity analysis. More than 13500 GEMs were generated by simultaneously varying the fraction of objective function and gene expression threshold linearly from 10^−6^ to 1, and between 1^st^ and 99^th^ percentile of the input expression profile, respectively.

#### iMAT

The existing implementation of iMAT (Shlomi method) in COBRA toolbox was used [15], and 3 parameters of the algorithm, namely, flux activation threshold (ε), low and high expression thresholds were selected based on sensitivity analysis. More than 14500 GEMs were generated by simultaneously varying the flux activation threshold (ε) from 10^−3^ to 10 (log-scale), low expression threshold from 1^st^ to 75^th^ percentile, and high expression threshold from 25^th^ to 99^th^ percentile.

#### mCADRE

The modified version of mCADRE available at https://github.com/jaeddy/mcadre was employed. In this version, fastFVA [33] was replaced by FASTCC [16] to accelerate the computations for checking the model consistency. All other parameters were left as their default values [12].

#### PRIME

The original implementation of PRIME [10] was employed, and TomLab solver was replaced by Gurobi Optimizer which was used for other *in silico* simulations.

#### INIT

The existing implementation of INIT in RAVEN toolbox was used here. INIT assigns weights to genes by dividing the gene expression in the target tissue to average expression across all the tissues [14]. Here, to be comparable with other algorithms, and due to the high correlation between cell lines in the NCI-60 panel (mean pairwise Spearman R = 0.91), the average gene expression across all cell lines were used in the weighting function.

#### FASTCORE

Since FASTCORE is a general algorithm for reconstruction of context-specific models, it does not introduce any assumptions for determining core reactions [16]. Therefore, core reactions were determined as described in the FASTCORMICS algorithm [11]. To this purpose, gene expression arrays (CEL format) were normalized with fRMA [34] via R-(D)COM Interface StatConnDCOM (http://www.statconn.com), processed with Barcode [35], and genes with z-scores above 5 were mapped to reactions using Gene-Protein-Reaction (GPR) rules to form the set of core reactions [11]. Finally, the biomass function was added to the core reactions.

#### FASTCORMICS

Identifying core reactions are similar to the procedure described for FASTCORE, with the difference that z-scores below 0 are considered as non-expressed [11]. As FASTCORMICS allows for the inclusion of biomass function along with the required reactions, this reaction was introduced to the algorithm and not independently added to the core reactions.

#### CORDA

Discretized z-scores used for FASTCORMICS were employed for CORDA: z-scores above 5 were considered for high confidence (HC) reactions, z-scores between 0 and 5 were considered for medium confidence (MC) reactions, and z-scores lower than 0 were considered as negative confidence (NC) reactions. Biomass function was also added to the set of high confidence (HC) reactions [19]. The constraint value for defining the reaction dependency was selected based on sensitivity analysis. Totally, 1200 GEMs were generated by linearly varying the constraint value from 1% to 99% of maximal flux rate.

#### TRFBA

The original implementation of TRFBA [18] was modified to reduce the computational cost. TRFBA adds two linear constraints to the algorithm, one for associating the reaction upper bounds with expression levels and the other for correlating the expressions of target and regulating genes. Here, to be compatible with other algorithms, we only used the first constraint. The parameter C was calculated with respect to the minimum growth rate error as reported in the original publication.

### Comparison analyses

#### Gene expression and growth rates

Processed and raw gene expression data for the NCI-60 panel were retrieved from Lee *et al* [36] and CellMiner database [37] using the same microarray panel (Affymetrix Human Genome U133A). Associated doubling times were obtained from the Developmental Therapeutics Program website (DTP) of the National Cancer Institute (https://dtp.cancer.gov/discovery_development/nci-60/cell_list.htm) and converted to growth rates (μ) by dividing ln(2) by the observed doubling times. The power of algorithms to predict the cancer growth was assessed by maximizing the biomass formation and the relative error was calculated as follows:

$$e=\frac{\left|growth^{\text{exp}}-growth^{pred}\right|}{growth^{\text{exp}}}$$ (2)

where *growth*^*exp*^ and *growth*^*pred*^ are observed and predicted growth rates, respectively.

#### Prediction of uptake/secretion rates

Metabolite uptake/secretion rates (CORE data) measured in Jain *et al* study [27] were normalized to the cell volume data measured by Dolfi *et al* [29] to evaluate the ability of generated GEMs to predict these experimental rates. According to the procedure described by Yizhak *et al* [10], the flux through the exchange reaction corresponding to the target metabolite was maximized under at least 90% maximum growth. Due to the removal of biomass function for iMAT, the output fluxes from the algorithm were used for comparison. Predictive power was determined by calculating the Spearman correlation and correcting the p-values for false discovery rate (FDR) using the Benjamini-Hochberg method (α = 0.05). When cell-specific medium was applied, uptake of the exchange reaction under test was set to its original value in the input human model to remove the constraining effect of measured metabolomics data on subsequent calculations.

#### Drug response simulation

According to the procedure introduced by Yizhak *et al* [10], enzymatic targets for the selected metabolic drugs [38–40] were obtained from DrugBank database [41], and their IC50 values (the required concentration of a drug for reducing the growth rate to 50% of its maximal value) were simulated by maximizing the flux through the target reaction and bounding the biomass to 50% of its maximum value. Predictive power of algorithms was evaluated by computing the Spearman correlation.

#### Prediction of cancer essential genes

Gene dependency scores (CERES) were retrieved from genome-scale CRISPR-Cas9 loss-of-function screen data publicly available in Project Achilles (Avana library, 18Q4 release) [42]. Briefly, CERES estimates gene dependency levels by accounting for the copy-number-specific effect, and therefore reduces false-positive dependencies [43, 44]. Here, genes with negative CERES dependency scores (<0) were defined as essential.

Flux balance analysis (FBA) was used to simulate the effect of gene knock-out on the growth rate. In accordance with previous studies [11, 25], genes which their knock-out reduced the maximal growth rate above 1% were considered as essential. A hypergeometric enrichment test was used to evaluate the predictive accuracy of the methods for 22 cell lines that were present in both Project Achilles and NCI-60 panel [42].

#### Prediction of oncogenes and tumor suppressors

A list of 903 oncogenes (OG) and 1247 tumor suppressor (TS) genes [45–47], along with a set of loss-of-function (LOF) mutations in several tumors which were suggested to be enriched with tumor suppressors [48] were collected. The enrichment of predicted OG, TS and LOFs was calculated by dividing the fraction of OG/TS/LOF in the set of predicted OG/TS/LOF by the fraction of OG/TS/LOF in the input general model [49]. The significance of the enrichment analysis was assessed using hypergeometric test. To observe the enrichment of generated GEMs with OG (higher is better) or TS/LOF (lower is better) genes, p-value calculations were carried out with respect to the right and left tail of the distribution for OG and TS/LOF, respectively [50].

### Consistency analyses

#### Network connectivity

The level of connectivity for each GEM was assessed by fast consistency evaluation method (*FASTCC*), which identifies reactions incapable of carrying a non-zero flux under any conditions (blocked reactions) due to the presence of dead-end metabolites or network gaps [16, 51]. The algorithms were then compared based on the mean fraction of blocked reactions present in the generated GEMs.

#### Similarity check

Jaccard similarity index was used to evaluate the degree of similarity among generated NCI-60 GEMs for each algorithm. To assess the ability of algorithms to distinguish between GEMs corresponding to specific cancer types, the average pairwise Jaccard index for GEMs associating with each cancer type in NCI-60 panel was computed, and the resolution power of examined algorithms were compared. Since algorithms were compared based on their reaction contents, only those capable of extracting a subnetwork from general human model were considered (i.e. all algorithms except TRFBA and PRIME).

#### Robustness analyses

Two different approaches were employed for robustness analysis: i) cross-validation to evaluate the confidence level of reactions included in generated GEMs [22], and ii) evaluate the robustness of algorithms to noise in the input expression data [21]. Due to computational difficulties, all analyses were only carried out for the GEMs generated for cell line RXF 393.

#### Cross-validation

Similar to the work of Pacheco *et al* [22], a repeated 5-fold cross-validation (for 15 times) was used by removing 20% of input core reactions at each time (i.e. a total of 75 GEMs). Hypergeometric test was applied to evaluate the capability of algorithms to return the removed reactions back to the generated GEM. For INIT and GIMME, 20% of reaction scores fed into the algorithm were set to 0 [22]. Since TRFBA and PRIME do not trim the generic model, cross-validation was performed by removing 20% of input expression data and evaluating the effect of missing expression data on growth rate prediction.

#### Robustness to noise

Gene expression data were randomly shuffled to generate a set of 20 noisy data (with same distribution of the original expression data) with similarly spaced intervals of Spearman correlation coefficients ranging from R < 0.004 for entirely random data to R = 1 for the original data [21]. These sets of random expression data were used to evaluate the impact of noise in the input data on the growth rate predictions. Furthermore, resolution power of generated GEMs regarding the noisy data was assessed by Jaccard similarity index.

## Results and discussion

The following 8 context-specific reconstruction algorithms were used in this study: GIMME, iMAT, INIT, mCADRE, FASTCORMICS, PRIME, CORDA and TRFBA (Table 1). These algorithms were originally developed or used in several studies to study cancer metabolic alterations, while FASTCORE, was also analyzed because it is the base algorithm for development of FASTCORMICS, and therefore, may be exploited in the future for investigations on cancer metabolism. Due to the high computational requirements of MBA, this algorithm was not included in current study despite its pioneer role in cancer metabolic modeling [25].

**Table 1:** An overview of the Context-specific reconstruction methods studied here.

Method	Approach	Input data	Subnetwork extraction	Optimization problem	Metabolic objective required
GIMME	Minimizes the inclusion of reactions with expression levels below a cutoff, while ensuring the activity of a defined objective function above a certain threshold.	Transcriptomic	Yes	LP	Yes
iMAT	Categorizes reactions into highly and lowly expressed reactions, and maximizes the consistency between reaction fluxes and corresponding expression states.	Transcriptomic	Yes	MILP	No
INIT	Assigns weights to reactions and maximizes the consistency between reaction fluxes and corresponding weights. Optionally, the accumulation of specific metabolites can be allowed.	Transcriptomic; Proteomic	Yes	MILP	No
mCADRE	Defines a set of core reactions based on network topology and expression data, and removes other reactions, while ensuring the activity of core reactions and a set of metabolic functions.	Transcriptomic	Yes	LP	No
FASTCORE	Extracts a consistent subnetwork consisting of all core reactions with addition of minimal number of non-core reactions.	Core reactions	Yes	LP	No
FASTCORMICS	A modified version of FASTCORE, with possibility of defining a set of non-penalized, and biomass supported reactions	Transcriptomic	Yes	LP	No
CORDA	Employs a dependency assessment by minimizing the flux through cost-consuming reactions. Only required undesirable reactions are included in the output network.	Core reactions	Yes	LP	No
PRIME	Uses the phenotypic data to identify phenotype-associated reactions, and modifies the bounds of corresponding reactions.	Transcriptomic; Phenotypic	No	LP	Yes
TRFBA	Uses phenotypic data to determine a constant to convert the gene expression levels to upper bounds of gene-associated reactions.	Transcriptomic; Phenotypic	No	LP	Yes

### Parameter optimization

Among all the methods studied here, GIMME, iMAT, CORDA and TRFBA rely on adjustable parameters. Hence, we evaluated the interaction effect of parameters on the generated GEMs, particularly on the growth rate prediction. Interestingly, both iMAT and GIMME tended to perform better in lower expression thresholds, fraction of objective function, and flux activation threshold (S1 Text). It is also of note that iMAT performed better when moderately expressed states were removed, and therefore, a single expression threshold was used. The constraint value of CORDA showed no evident effect on the growth prediction (S1 Text). Finally, the constant parameter C in TRFBA exhibited a non-monotonic dependence on growth rate prediction error as previously reported (S1 Text).

### Comparison-based analyses

#### Phenotypic analyses

**Fig 1:**
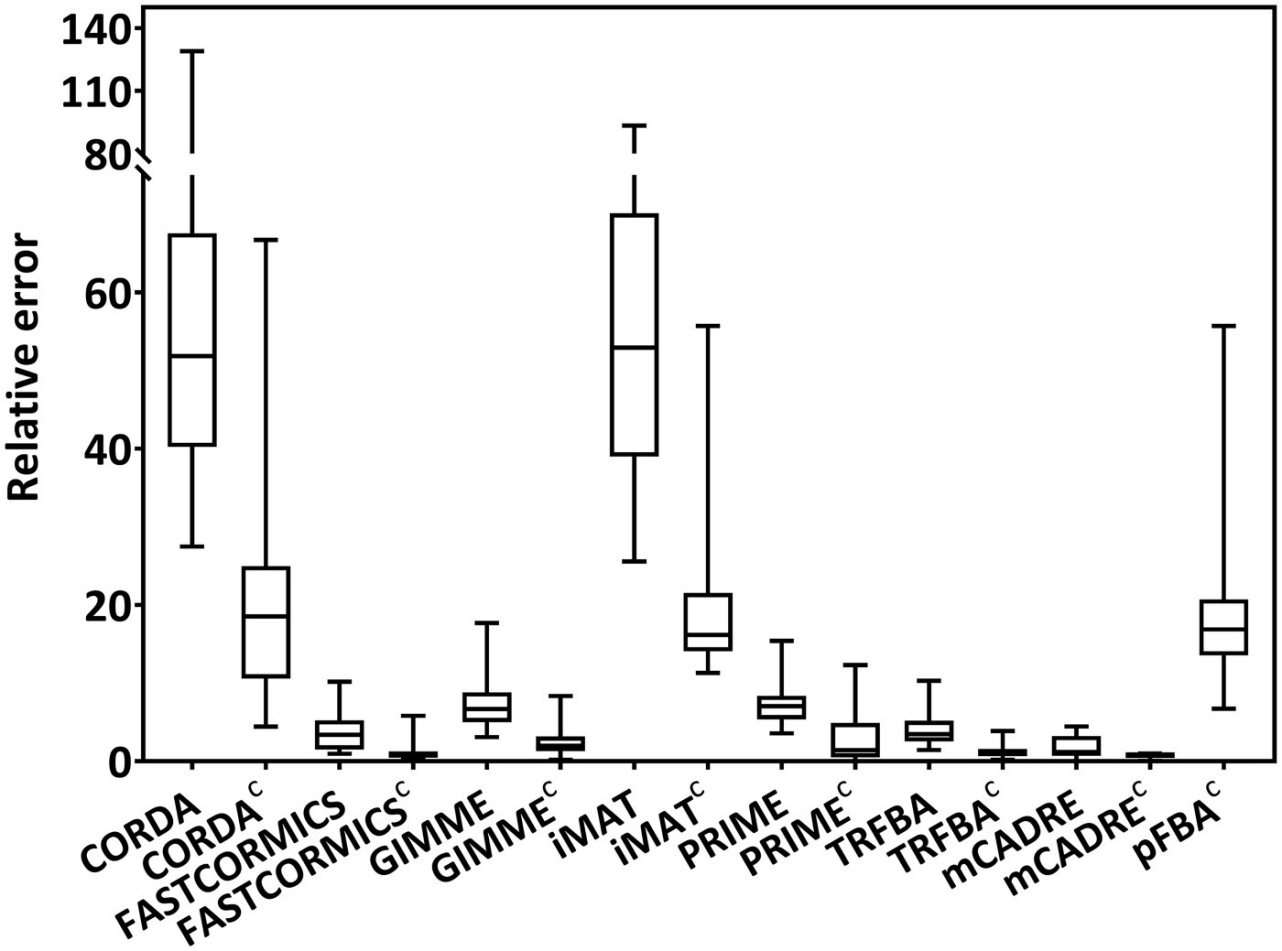
Growth rate prediction. Distribution of relative error for prediction of growth rates for all algorithms using both general and cell-specific medium (designated by the superscript c). Each box-plot shows the distribution of error across all cell lines in NCI-60 panel. Only algorithms capable of predicting non-zero growth rates were depicted. Relative error was calculated according to the (Eq 2).

Due to the heterogeneity of the data exploited by different context-specific modeling algorithms, it is of paramount importance to provide a general platform consisting of all these experimental data sets to compare the predictive power of existing or newly developed computational methods. To evaluate the capability of each algorithm to estimate the growth rate of cancer, relative error was calculated based on observed and predicted growth rates (Fig 1). Conceivably, the GEMs generated with cell-specific medium (denoted with c superscript) showed a better performance compared to their counterparts with general medium. Both CORDA and iMAT exhibit similar behavior, and a worse ability to predict growth rates. This may be due to the addition of a biomass reaction to the high confidence reaction set in CORDA, forcing the algorithm to include reactions from medium and negative confidence reaction sets within the final GEM. Moreover, FASTCORMICS and mCADRE use a set of core reactions and extract a subnetwork from input generic model while trying to include the core set in the final GEM. Although both algorithms show a low error distribution, not all the generated GEMs were capable of predicting the growth *in silico* (25% and 17% for FASTCORMICS and FASTCORMICS^C^, respectively, and 41% and 39% for mCADRE and mCADRE^C^, respectively). Among all algorithms, TRFBA exhibits superior capability to predict cancer growth; however, this is not surprising since TRFBA employs an optimized constant based on prior knowledge of observed growth rates [18].

**Fig 2:**
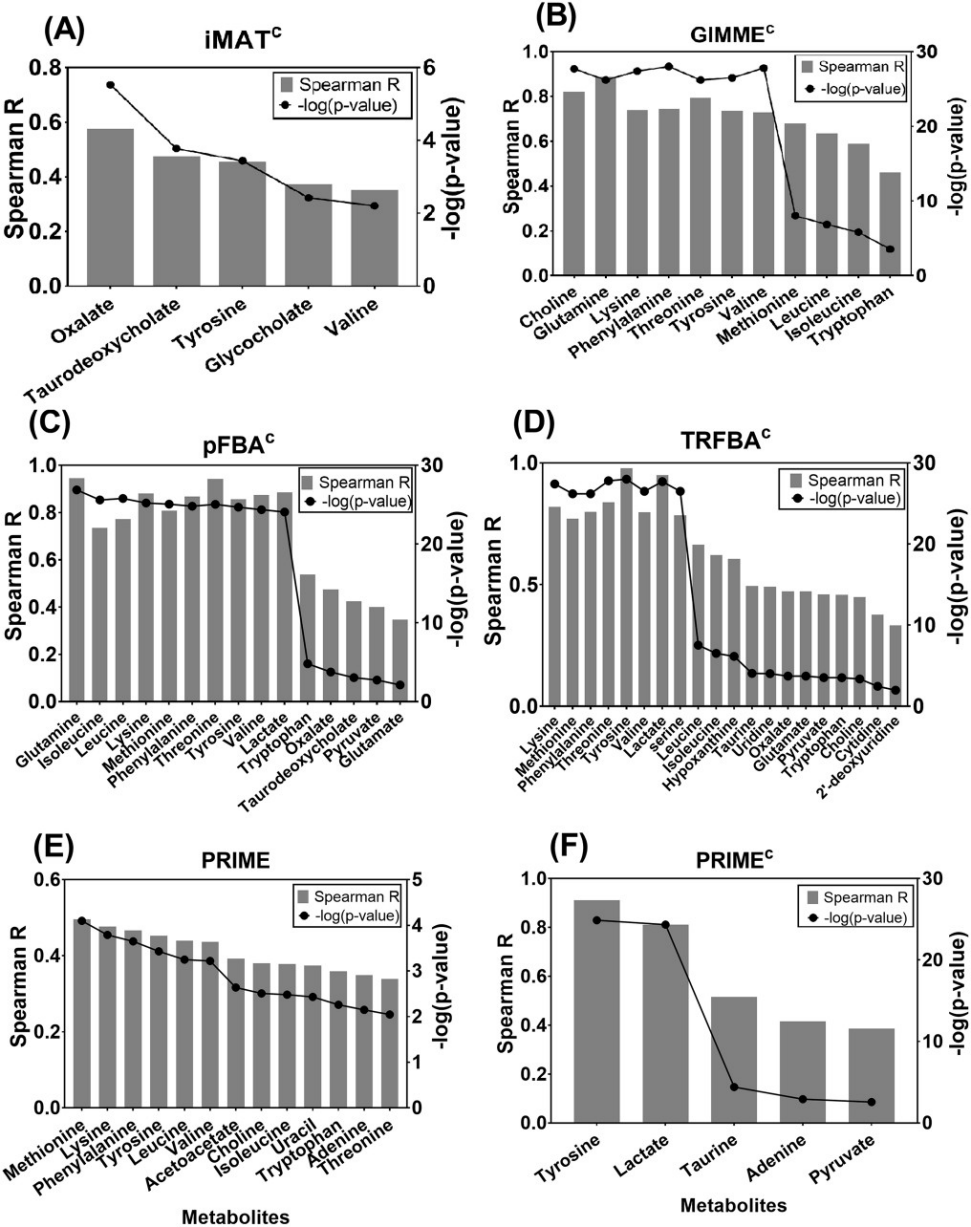
Uptake/secretion rates prediction. Spearman correlation between measured and predicted uptake/secretion flux rates of metabolites for (A) iMAT^c^, (B) GIMME^c^, (C) pFBA^c^, (D) TRFBA^c^, (E) PRIME, and (F) PRIME^c^. Represented p-values were adjusted for False discovery rate (α = 0.05). Only methods with significant predictions are shown.

Next, to compare the algorithms based on their ability to predict observed uptake/secretion rates, we used exometabolomics data for metabolites with an exchange reaction present in the input model. Only 5 algorithms (Fig 2) resulted in significant correlations, among which pFBA^c^ only employed cell-specific medium as constraint. Apart from PRIME, which was capable of generating significant predictions using general medium, other significant predictions were the result of constraining the GEMs with cell-specific medium, showing the key role of metabolomics data in promoting the prediction accuracy of intracellular fluxes [52]. It is of particular note that PRIME predicted a wider range of uptake/secretion rates than PRIME^c^. PRIME uses phenotypic data (e.g. observed growth rates), and by using a correlation based approach, tries to find the genes with expression levels that are significantly correlated with growth rates across the studied cell lines (e.g. NCI60 panel). As the result, the growth associated reactions identified by this method are independent of the input constraining criteria (i.e. cell-specific or general media). However, flux bounds of growth associated reactions depend on the min/max range, which in turn are affected by constraining criteria. Therefore, it seems that the modified upper bounds in RPIME are less consistent with the objective functions when constrained with cell-specific medium. In this case, the algorithm may experience the over-constrained situation which may explain the poorer performance of PRIME^c^ compared to PRIME (as shown in the following sections).

Notably, three algorithms (Fig 2C, 2D and 2F) showed a strong correlation (Spearman R > 0.8) between predicted and measured lactate secretion rates. This is of special interest because elevated lactate secretion is a major hallmark of cancerous cells [53], and attempts have been made to predict meaningful lactate flux rates in the context of cancer metabolic modeling [2, 54]. It is also of note that both TRFBA and PRIME adjust the flux bounds of generic input model using prior knowledge of observed growth rates, while other algorithms studied here try to extract a fixed subnetwork from the general human model [20]; however, GIMME adopts an inclusive reconstruction approach, which may explain the ability of GIMME^c^ to predict metabolite uptake/secretion rates.

**Fig 3:**
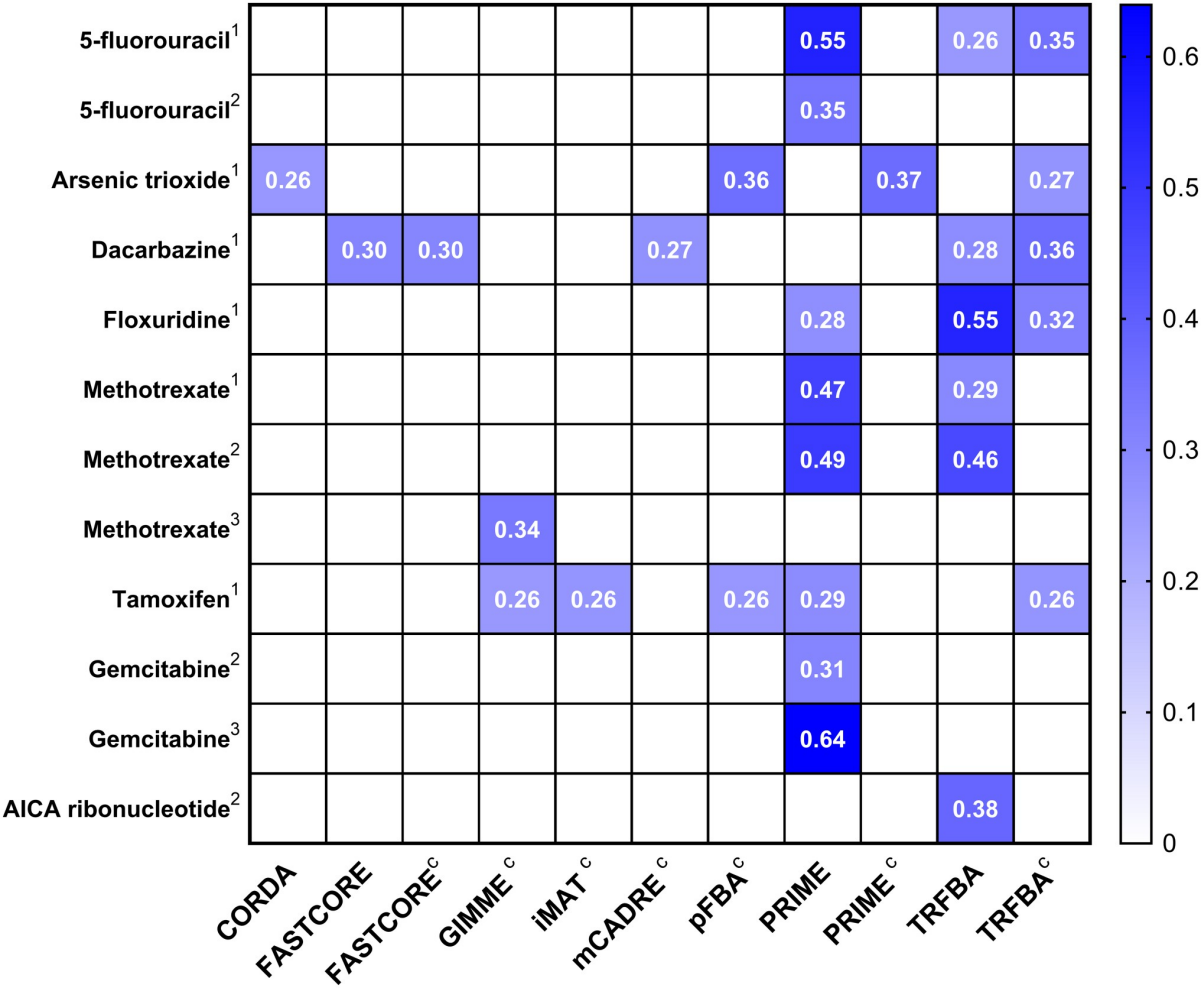
Drug response predictions. Heatmap of significant Spearman correlations between simulated and experimental drug response data. The Spearman coefficients for each drug have been shown on the figure. Superscripts indicate drug response data taken from (1) Holbeck *et al* [38], (2) Garnett *et al* [39] and (3) Yang *et al* [40]. Only methods with significant drug responses are shown.

All algorithms were also investigated for their ability to predict drug response based on the approach described by Yizhak *et al* [10] (see Methods for more details). As depicted in Fig 3, while the correlation between the predicted and measured drug responses for most of the algorithms is weak, PRIME and TRFBA predicted a wider range of drugs with slightly stronger correlations. Furthermore, both PRIME and TRFBA outperformed their cell-specific counterparts. We therefore examined the flux distributions of both algorithms for Methotrexate (both PRIME and TRFBA constrained with cell-specific medium failed to predict its drug response). Our analysis showed that compared with general medium conditions, more exchange reactions constrained with cell-specific medium reached their upper limits. Especially in the case of PRIME^c^, many of the internal reactions for which the bounds were adjusted, reached their limits, suggesting that the solution space was shrunk due to these governing constraints.

Again, GIMME^c^ was able to identify significant correlations between predicated and measured IC50 values for two different drugs (Tamoxifen and Methotrexate). Although most of the resulting Spearman correlations for drug response simulations (as a proxy of internal fluxes of the network) were weak, the use of cell-specific medium had a modest positive effect on the performance of GIMME, iMAT, mCADRE and pFBA. Additionally, while cell-specific constraints reduced the predictive power of TRFBA and PRIME, incorporating observed growth data into the reconstruction pipeline of these approaches, improved their phenotypic prediction performance over their competitors [10, 18].

#### Genotypic analyses

**Fig 4:**
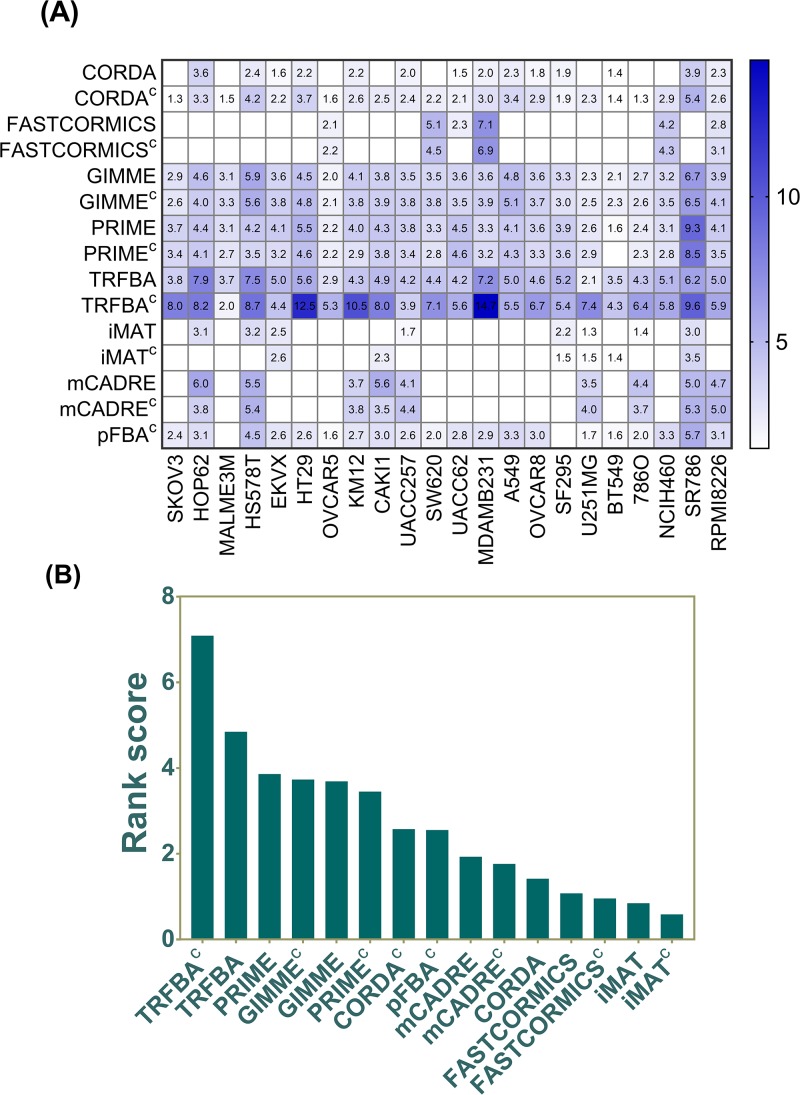
Prediction of general cancer essential genes. (A) Heatmap of enrichment p-values for predicted cell-line specific essential genes. The numbers indicate -log_10_ enrichment p-values. GEMs with insignificant p-values are shown in white. (B) Rank scores of the algorithms based on their significance and the number of GEMs with significant enrichment (as described in S2 Text).

Since identifying novel therapeutic targets is another important aspect of cancer metabolic modeling [2, 3, 9], the algorithms were also evaluated based on their ability to predict cancer essential genes [42]. The mean enrichment p-values (log-transformed) and the fraction of significant cell lines (of 22) per algorithm, were used to rank the performance of each method (Fig 4B, S2 Text). While, the cell line-specific models generated by TRFBA, PRIME and GIMME were more enriched (Fig 4A) in cell line-specific essential genes, low performance of other algorithms are mainly due to their inability to reconstruct functional GEMs for a number of cell lines. Importantly, similar to the findings observed with metabolite uptake/secretion rates and drug response simulations (Figs 2 and 3), the incorporation of cell-specific medium showed a double-edged effect on different algorithms. On one hand, it markedly improved the predictive capability of TRFBA by modulating the upper bounds of reactions supported by metabolic genes. On the other hand, it negatively affected the performance of PRIME (Figs 2, 3 and 4), which shares similar characteristics to TRFBA (Table 1). One important difference between the two methods is the definition of normalization range by PRIME, which acts as additional constraints to narrow down the solution space [10]. The relatively lower uptake fluxes within cell-specific medium compared to those in general medium, further tighten the constraints imposed on PRIME^c^, which may explain its poorer performance compared with TRFBA^c^ or PRIME with looser constraints. Moreover, TRFBA uses the expression levels to limit the rate of a subset of reactions associated with a certain gene, rendering the model more flexible compared with fixed upper bounds used by PRIME [18].

Moreover, since oncogenes and tumor suppressors are involved in conferring malignant phenotype to tumor cells [55], it is of great importance to evaluate context-specific algorithms for the number of oncogenes and tumor suppressor genes [45]. In addition, as mutational activation of oncogenes (OG) and loss of function (LOFs) mutations of tumor suppressors (TS) are of pivotal importance in cancer progression [55], higher and lower enrichment of these mutations respectively may denote higher context-specificity of assessed algorithms.

**Fig 5:**
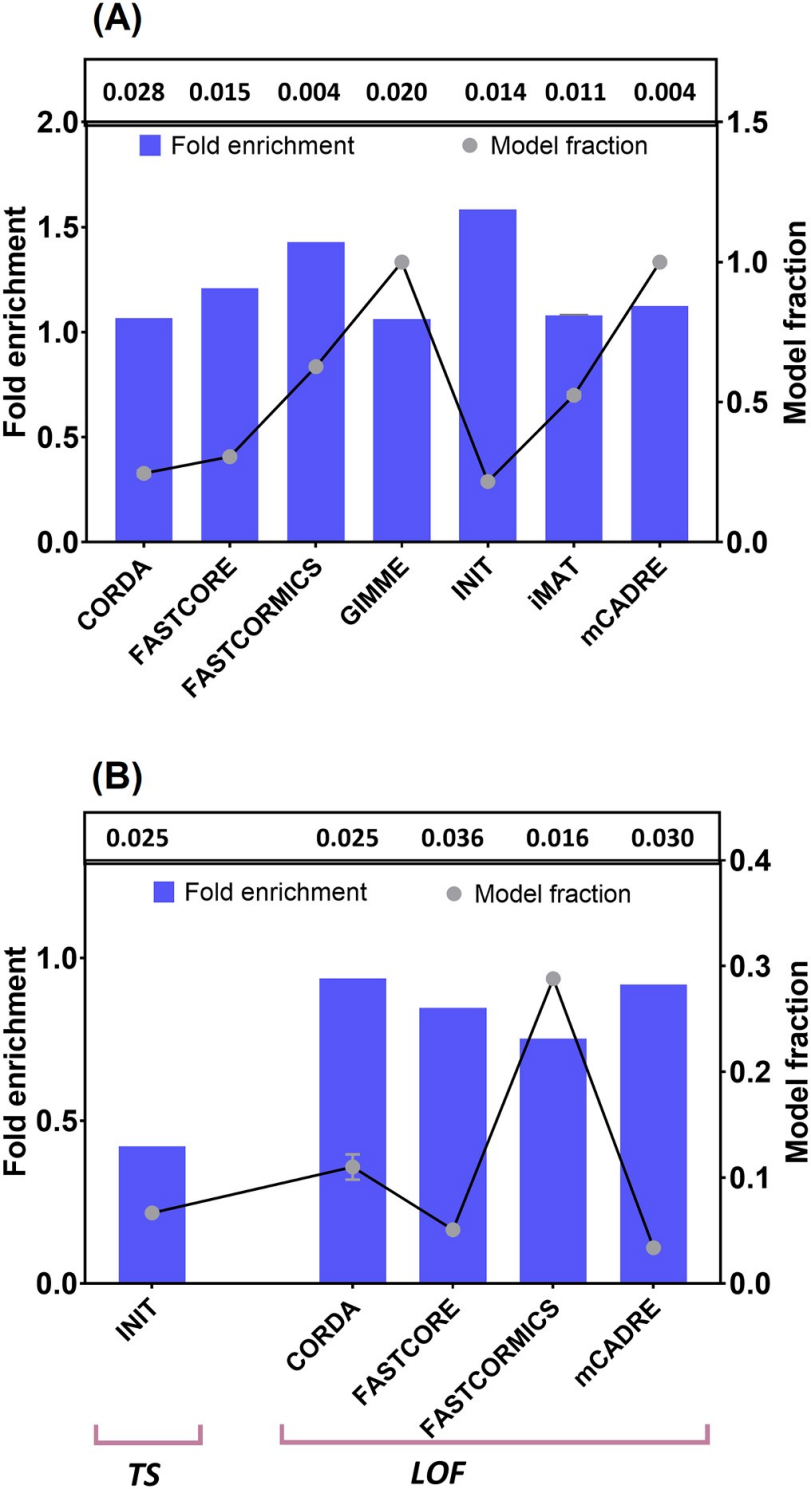
Prediction of oncogenes (OG), tumor suppressors (TS) and loss of function (LOF) mutations. Mean enrichment of predicted (A) OGs and (B) TS and LOFs with experimental data. The error bars show the standard deviation across GEMs generated with general and cell-specific media. Hypergeometric p-values are shown above each figure. Model fraction represents the fraction of generated GEMs with significant p-values (<0.05). Only methods with significant predictions are shown.

Although INIT showed higher enrichment values (higher for OGs, and lower for TS and LOFs), the fraction of significant models was low (Fig 5). However, in terms of both fold enrichment and model fraction, FASTCORMICS displayed a relatively better predictive performance compared to other methods. It is also of note that employing cell-specific medium (using exometabolomics data) had little or no effect on the number of OG, TS and LOFs included in the final GEMs (Fig 5). This may be due to the construction pipeline of some algorithms (FASTCORE, FASTCORMICS and mCADRE), which select core reactions without taking into account the influence of media constraints [11, 12, 16], or use experimental data (INIT) to assign weights to gene-associated reactions [14]. On the other hand, employing FBA in GIMME, iMAT and CORDA as part of their construction process may explain little variation between the generated GEMs (Fig 5).

### Consistency-based analyses

**Fig 6:**
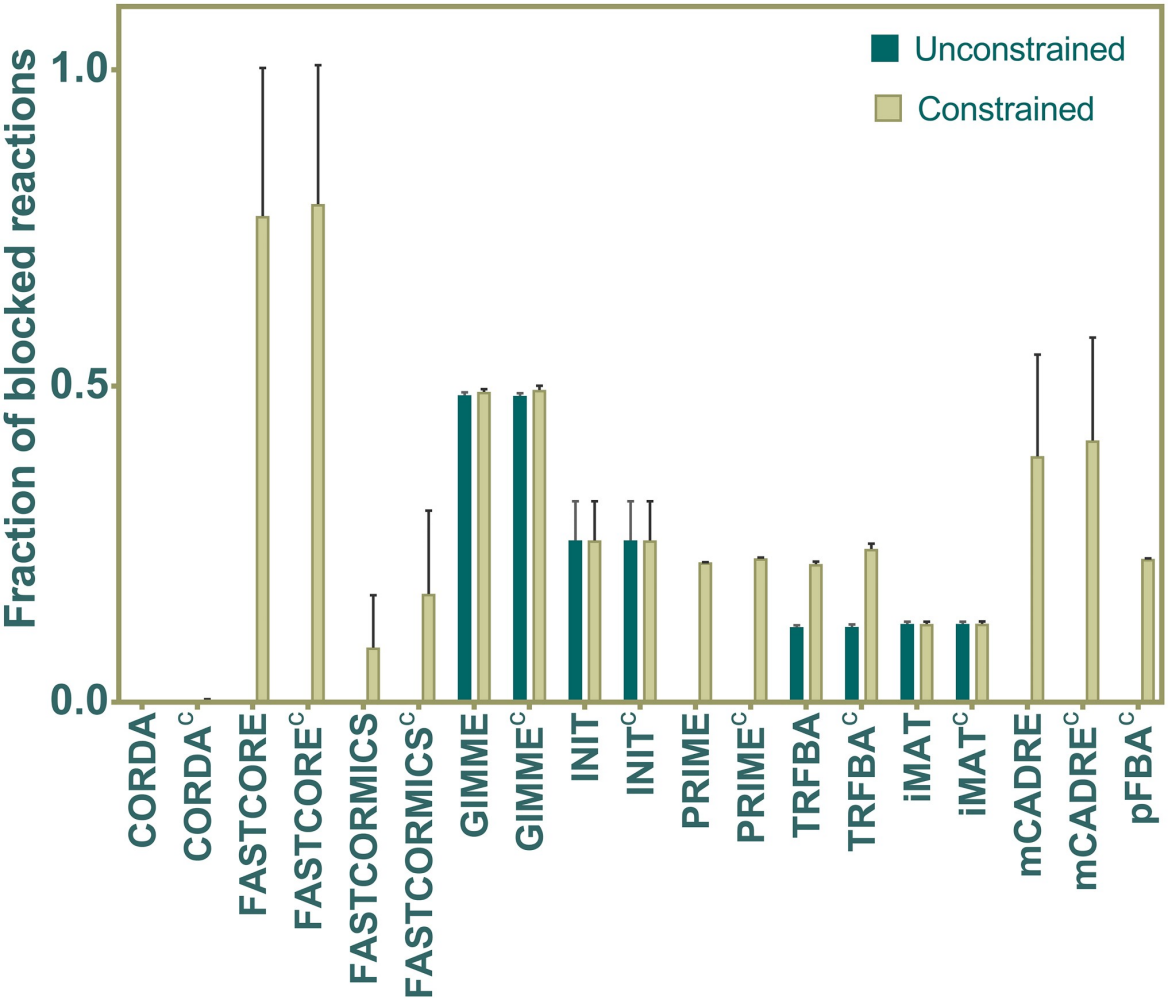
Network connectivity of generated GEMs. The fast consistency evaluation method [16] was used to identify the fraction of blocked reactions in the GEMs reconstructed by each method. The presence of blocked reactions were assessed in both constrained and unconstrained states. Data shown as mean fraction of existing blocked reaction across all generated GEMs, and error bars represent the standard deviation.

Consistency tests used here are mainly built on the approaches adopted by Pacheco *et al* [22] and Machado and Herrgård [21]. We were particularly interested in studying the properties of the generated GEMs regarding their topological holes (the extent of blocked reactions), capability to differentiate between different contexts (e.g. tissues or cell types), and their robustness to the missing or noisy data in the input. Since the flux consistent part of Recon 1 was employed here (i.e. all blocked reactions were removed prior to the simulations), we evaluated the ability of each algorithm in generating connected networks in both constrained and unconstrained states (Fig 6). There exist a number of methods for which the constraining criteria had a large effect on the fraction of blocked reactions. It is of note that both FASTCORE and FASTCORMICS share similar fundamental properties; however, the lower number of blocked reactions in FASTCORMICS may be ascribed to the inclusion of biomass supported reactions and defining non-penalized reactions [11]. Most notably, GIMME contained the highest fraction of blocked reactions in unconstrained state. Since the expression threshold used here for GIMME was relatively small, there were a large number of reactions considered to be active while not supported by growth. Therefore, the algorithm favored their inclusion, while they became blocked due to removal of unexpressed reactions. Both PRIME and TRFBA share a similar number of blocked reactions in the constrained state, which presumably was the result of bound constraints.

**Fig 7:**
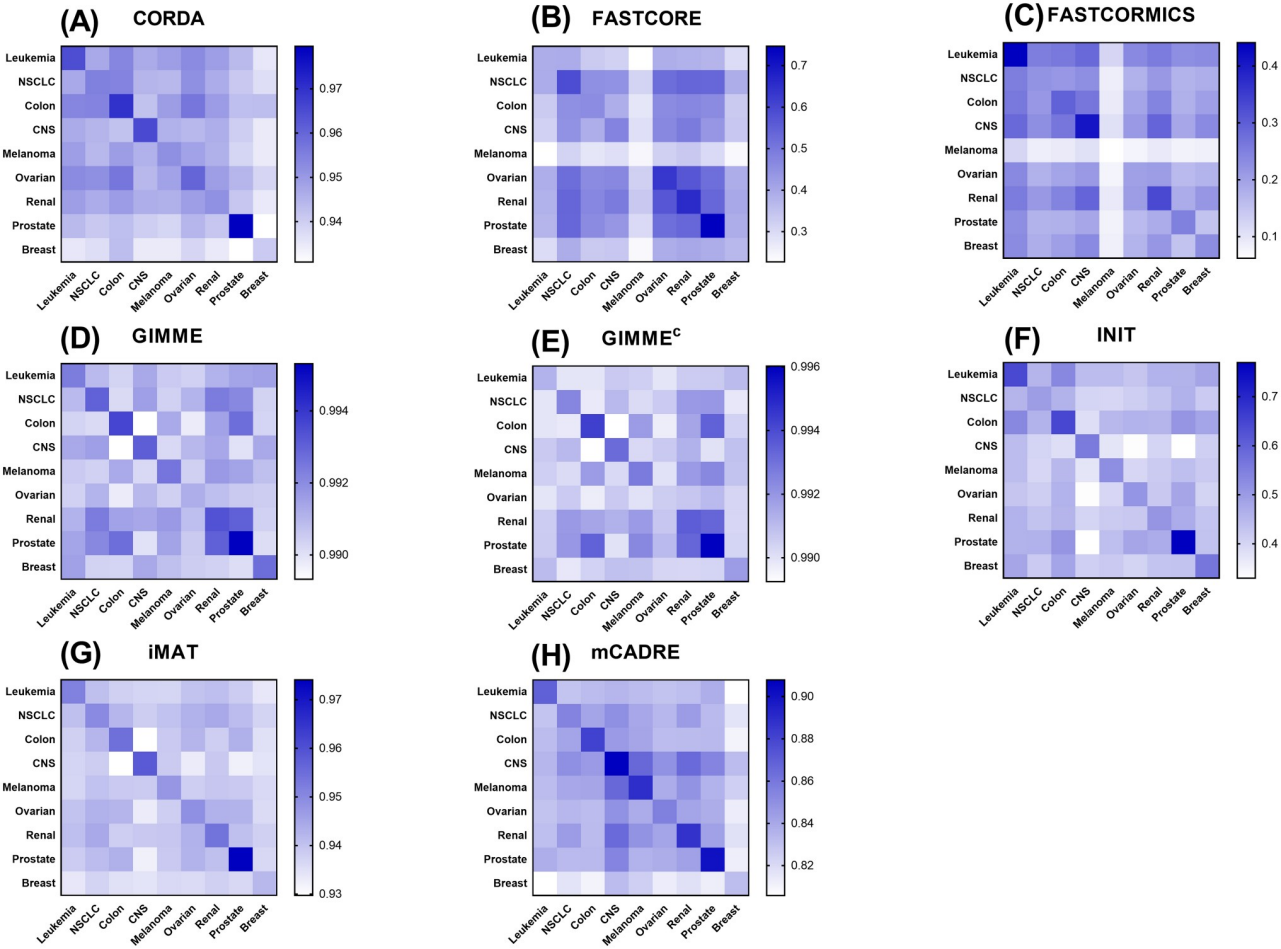
Similarity levels of generated GEMs between different tumors. Average Jaccard similarity index computed for GEMs built by (A) CORDA, (B) FASTCORE, (C) FASTCORMICS, (D) GIMME, (E) GIMME^c^, (F) INIT, (G) iMAT, and (H) mCADRE. Each square represents the average pairwise Jaccard value for each cancer type in the NCI-60 panel.

Next, the average Jaccard similarity index was calculated across different tissue types in the NCI-60 panel to evaluate the ability of the methods to distinguish between distinct cancer types (Fig 7). Considering the high correlation between expression data used here (pairwise Spearman correlation coefficient range: 0.87–1), this assessment provides a useful basis for comparing resolution power of different algorithms.

The diagonal in Fig 7 represents the level of similarity between GEMs generated for a particular type of cancer. Hence, it is expected that algorithms with higher resolution power result in heat maps with high similarity among models of a certain tumor type (dark blue), while the similarity among other cell-specific models remains lower (light blue). We devised a scoring scheme (S2 Text) to quantitatively compare the similarities between the GEMs coming from each algorithm. INIT, which maximized the consistency between expression profile and model reaction fluxes exhibited comparatively better resolution powers (Fig 7F), followed by FASTCORE and FASTCORMICS (Fig 7B and 7C). Notably, while FASTCORMICS was developed based on the FASTCORE, their ability to generate tissue-specific models was not similar (e.g. prostate, ovarian and renal models in FASTCORE, and leukemia and CNS in FASTCORMICS in Fig 7B and 7C). Furthermore, although CORDA and mCADRE share a similar reconstruction pipeline in terms of selecting a set of core and non-core reactions, mCADRE showed a relatively better resolution power, which may be due to the use of a so-called flexible set of core reactions by mCADRE, which in turn improved its tissue specificity [12]. Moreover, looking at the tissue specificity of CORDA and GIMME (Fig 7A and 7D), it appears that the overall similarity across the tissue-specific GEMs is considerably high (Jaccard index range of 0.93–0.98 for CORDA and > 0.99 for GIMME), indicating the inclusive approach of the two algorithms. In addition, as observed for the enumeration of OG, TS and LOFs, cell-specific media had little or no influence on the context-specificity of most algorithms (except for GIMME^c^ in Fig 7E).

Next, the robustness of GEMs to missing data in the input expression profile was evaluated using 5-fold cross-validation. As shown in Table 2, only INIT and FASTCORMICS were significantly able to recover the missing input reactions to the final GEMs.

**Table 2:** Cross-validation test results for the context-specific algorithms under study.

Algorithm	Input model	GEM size	Recovered reactions	Validation set	Hypergeometric p-value
INIT	2473	282.9 (22.4)	91.6 (11.1)	191.6 (0.5)	2e-40
FASTCORMICS	2473	440.5 (9.7)	12.8 (2.5)	39	0.03
CORDA	2473	1797.3 (4.1)	25.9 (3.2)	39	0.91
FASTCORE	2473	639 (41.3)	12.3 (2.8)	39	0.29
GIMME	2473	1126.3 (7.3)	11.8 (3.8)	260.4 (0.5)	0.99
iMAT	2473	2021.1 (13.6)	134.1 (7.1)	206.6 (0.5)	0.99
mCADRE	2473	1318.6 (26.4)	55.8 (5.3)	209	0.99

Data are presented as mean and standard deviation in parenthesis.

**Fig 8:**
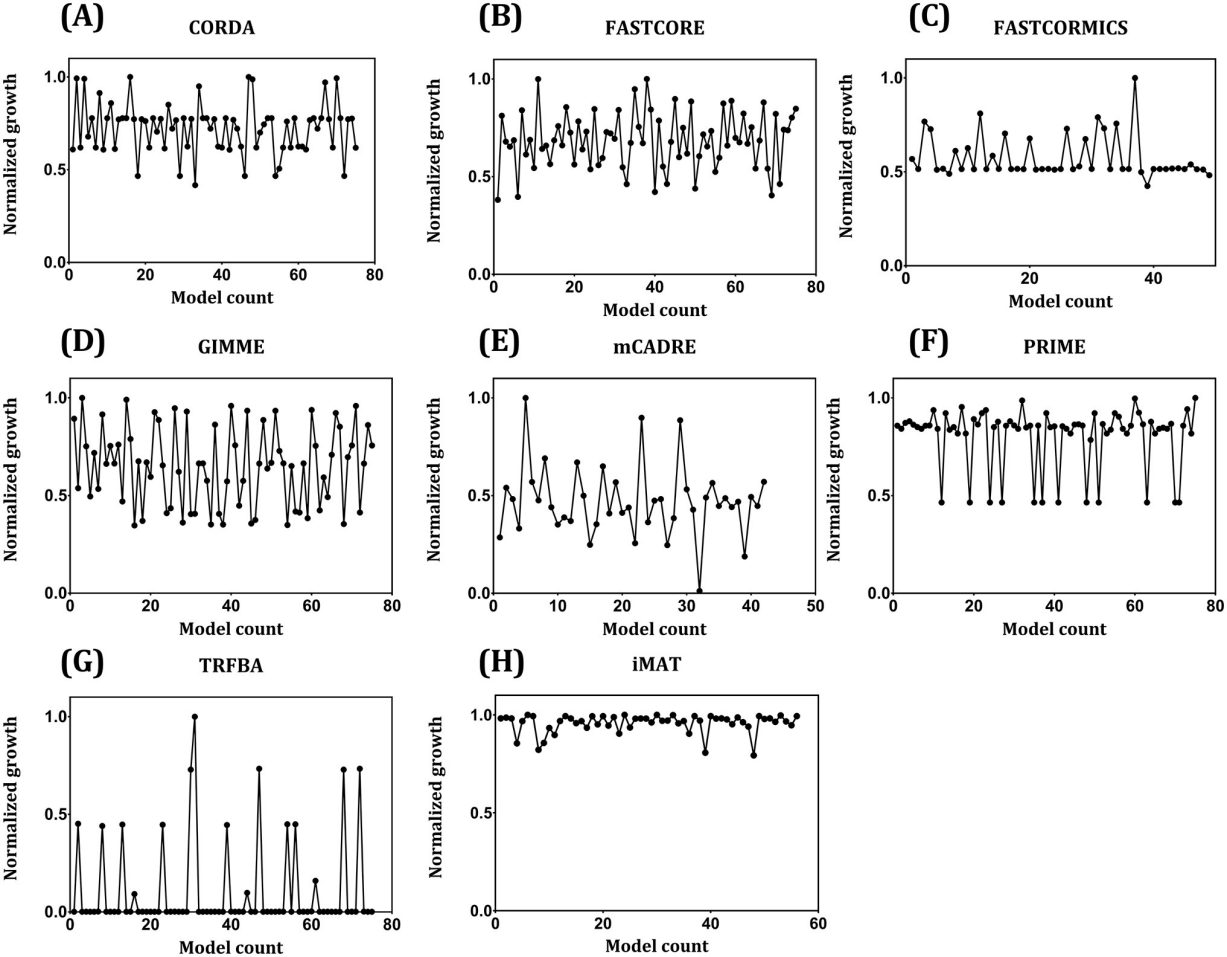
Normalized growth prediction of GEMs generated using data from repeated 5-fold cross-validation. (A) CORDA, (B) FASTCORE, (C) FASTCORMICS, (D) GIMME, (E) mCADRE, (F) PRIME, (G) TRFBA, (H) iMAT. Only algorithms capable of predicting growth are shown. For each algorithm, “model count” represents the GEMs generated by incomplete expression data or core reactions set in the input. For a better comparison, growth rates were normalized to the maximum value.

Furthermore, the robustness of algorithms capable of predicting a non-zero growth rate was further evaluated to the missing input data (Fig 8). iMAT exhibited a robust behavior in growth rate predictions (less variation among different sets of input reactions), which may be attributed to its focus on flux consistency maximization rather than on the growth rate. Therefore, the missing reactions in the input affected the content of the network and not the biomass supported reactions (Table 2). Moreover, the behavior of FASTCORMICS, PRIME and TRFBA are similar, with few variations among different validation sets (Fig 8C, 8F and 8G). Lastly, CORDA, FASTCORE and mCADRE were less robust to different input reaction sets (Fig 8A, 8B and 8E).

**Fig 9:**
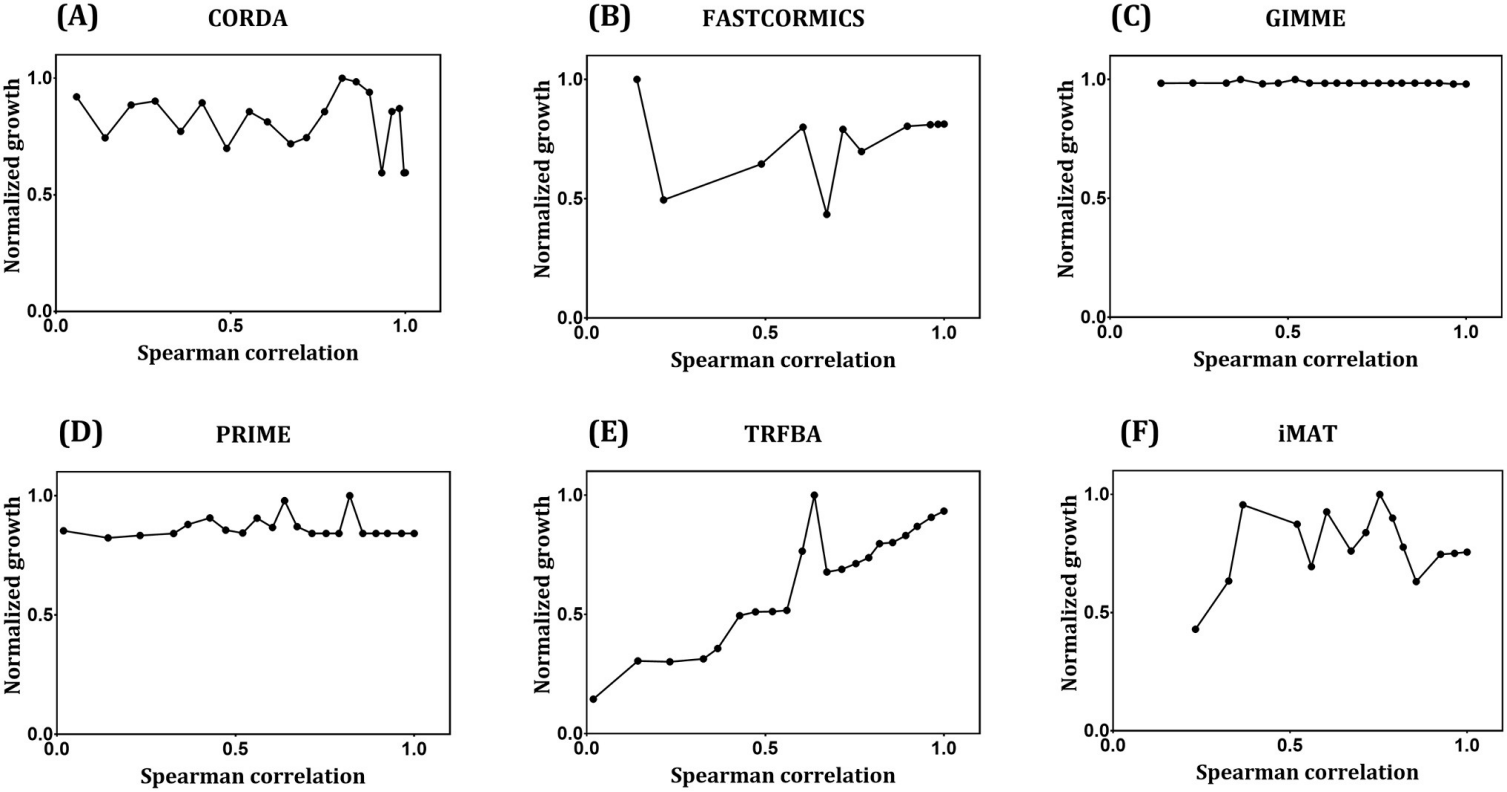
Normalized growth prediction of GEMs generated using noisy expression data. (A) CORDA, (B) FASTCORMICS, (C) GIMME, (D) PRIME, (E) TRFBA, and (F) iMAT. Only GEMs capable of predicting growth are shown. The x-axis shows the spearman correlation coefficient between each set of noisy data and original expression profile ranging from 1 (original) to R < 0.004 (random). For a better comparison, growth rates were normalized to the maximum value.

**Fig 10:**
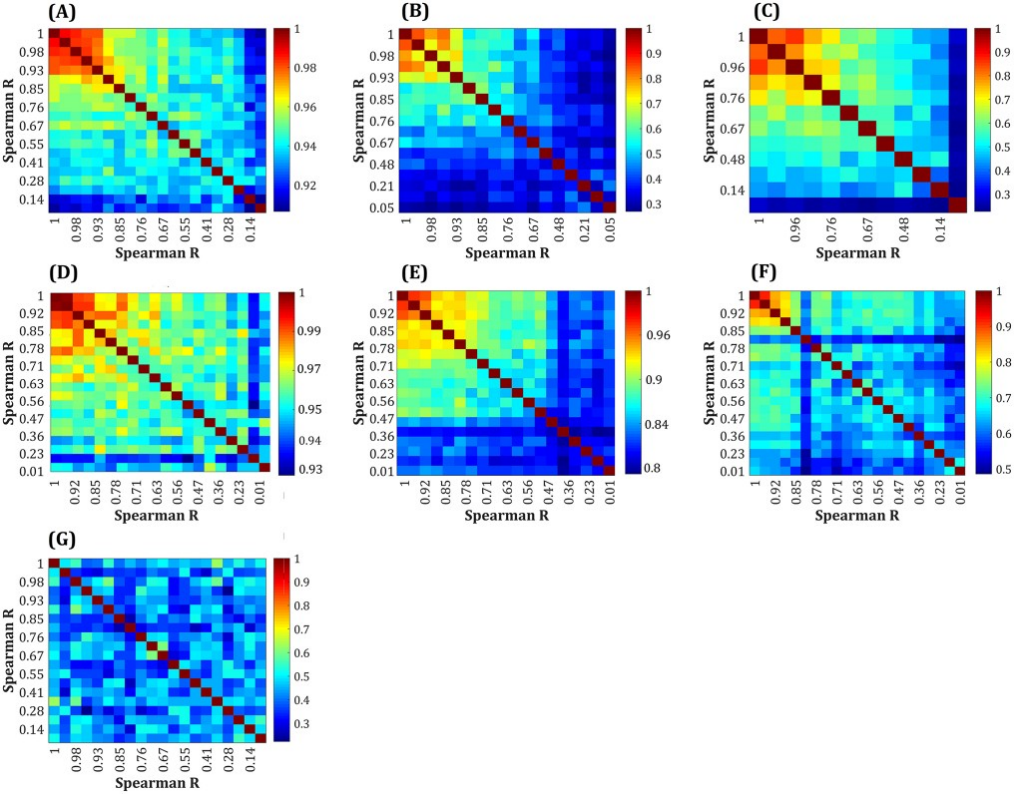
Similarity levels of GEMs generated with different sets of noisy expression data (A) CORDA (B) FASTCORE (C) FASTCORMICS (D) GIMME (E) iMAT (F) mCADRE (G) INIT.

To examine the robustness of algorithms to noise in the gene expression data, original expression profile was shuffled to introduce increasing levels of noise ranging from original data (Spearman R = 1) to completely shuffled data (Spearman R ~ 0). Normalized growth rate predictions of GEMs generated with these sets of noisy data are shown in Fig 9. Although robustness to noise is considered as an advantage of context-specific algorithms, the algorithms should be also able to distinct between similar expression patterns [10, 21, 22]. Hence, it is expected that a powerful algorithm in this context shows a moderate variation in flux predictions/network content at lower noise levels, with higher variations at higher noise levels. This behavior can be clearly seen for FASTCORMICS and iMAT, and to a certain extent for TRFBA (Fig 9B, 9F and 9E). However, the noise threshold for such a behavior seems to be context-dependent. The resolution power of these algorithms shown in Fig 10, provides a further examination of the impact of noise on network structure of generated GEMs. As can be seen, when noise level is low (high correlation coefficients), FASTCORE and FASTCORMICS generated structurally similar models. These algorithms however gained their ability to distinguish among expression patterns at high noise levels (Fig 10B and 10C). CORDA and GIMME showed a similar behavior to what was observed with similarity levels of tumor GEMs with highly similar networks (Jaccard index range of 0.93–1 for GIMME and 0.91–1 for CORDA) across different noisy data (Fig 10A and 10D). Furthermore, iMAT and mCADRE resulted in relatively similar response to noise in the input data, with a gradual transition from similar to distinct networks (Fig 10E and 10F). Most notably, INIT robustness to the introduced noise was comparably low. Although this may explain the satisfactory performance of the algorithm in differentiating the tumor GEMs (Fig 7F), the ability of algorithm to generate distinct networks from similar expression data (e.g. in different stages of cancer progression) remains unclear.

### Benchmark-driven approach

**Fig 11:**
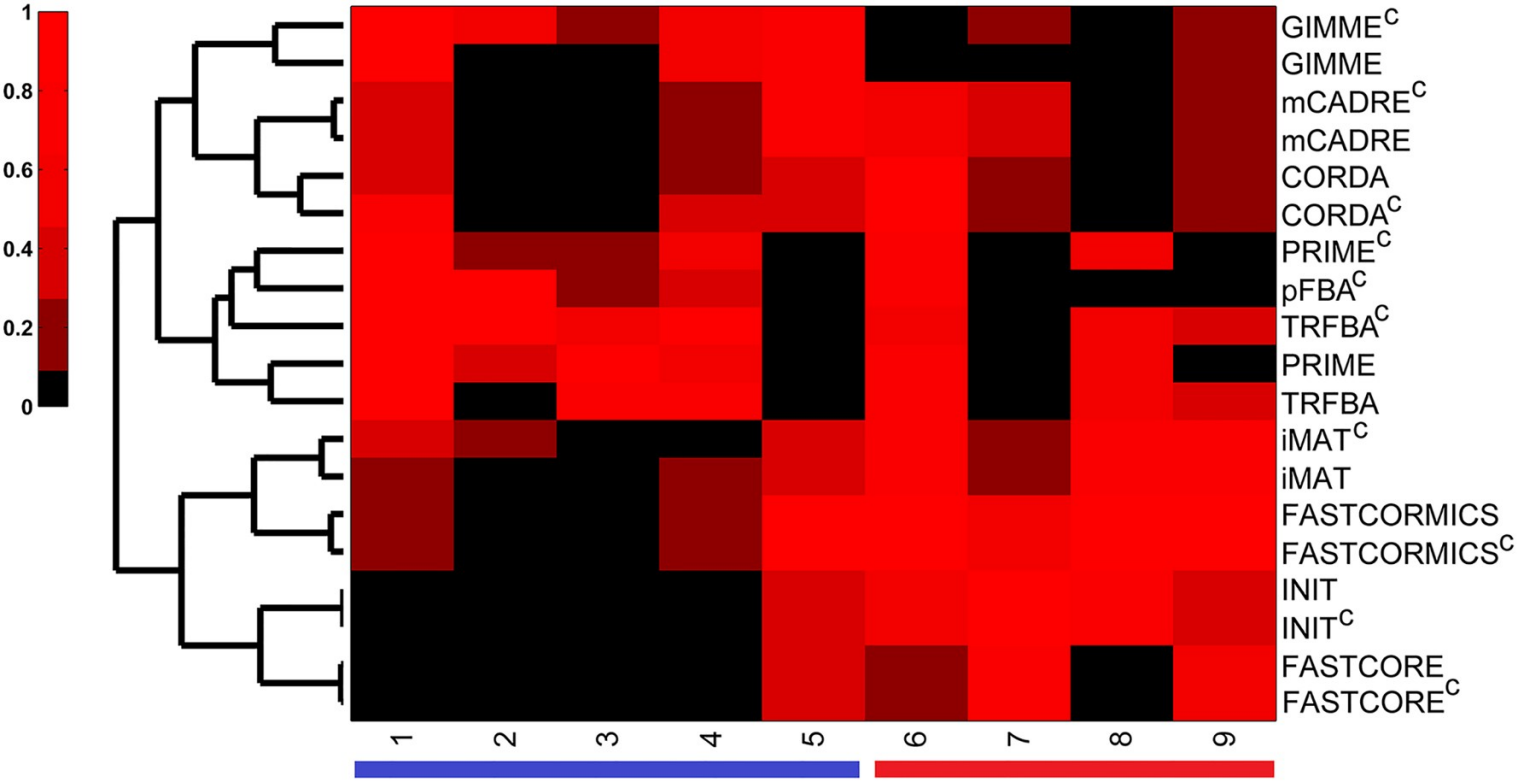
Benchmark performance scores for algorithms under study. Hierarchical clustering (Euclidean distance) of the scores each method received over different benchmarks. Three main clusters were identified: 1- GIMME, CORDA and mCADRE with an overall weak to moderate performance; 2- PRIME, TRFBA and pFBA^c^ with strong performance in comparison tests, and 3- FASTCORE, INIT, iMAT and FASTCORMICS with strong performance in consistency tests. Numbers in column correspond to comparison (blue color) or consistency (red color) benchmarks: 1-growth rate, 2- metabolite uptake/secretion rates, 3- drug response, 4- essential genes, 5- enrichment of OG/TS/LOFs, 6- fraction of blocked reactions, 7- resolution power, 8- robustness to missing data, and 9- robustness to noise. Colorbar indicates normalized performance scores.

Both comparison and consistency tests employed here were based on previous cancer metabolic modeling studies, and can be served as a guideline for selecting the best algorithmic approach for the study of specific aspects of cancer metabolism [10, 11, 22]. While several context/cell/tissue-specific algorithms have been developed so far, and their numbers are expected to grow in future, there are few reports on developing algorithms based on already existing context-specific algorithms (e.g. FASTCORMICS, MPA and tINIT based on FASTCORE, iMAT and INIT, respectively) [11, 56, 57]. Furthermore, none of these methods were developed as the result of thorough examination of other existing algorithms. Thus, providing an appropriate phenotypic and consistency benchmark for algorithms used in cancer metabolic modeling is not only important for selecting the most accurate algorithms, but it may also play a role in designing and developing new algorithms that best recapitulate the underlying metabolic dysregulation in cancer. Here, we devised a quantitative scoring scheme to provide a basis for evaluating various aspects of algorithms under study (S2 Text). We next hierarchically clustered the resulting scoring matrix to classify the methods based on their performance in different benchmarks (Fig 11).

The methods were clustered into three major groups. Group 1 contains 3 methods, with 2 of them (CORDA and mCADRE) trying to generate a functional GEM comprising of a set of pre-defined core reactions. Unlike FASTCORE and FASTCORMICS, they are pruning algorithms, and do not intend to generate a minimal GEM, but rather a functional one, which may explain their relative closeness to GIMME, the third algorithm in the group 1. Overall, the 3 algorithms showed a weak to moderate performance over all benchmarks, which can be attributed to their inclusive approach. Algorithms in group 2 retain the general human network, while tuning the solution space by relying on the prior knowledge of phenotypic data (growth rate). Interestingly, while the methods constrained with cell-specific medium grouped together in small sub-clusters, TRFBA^c^, pFBA^c^ and PRIME^c^ were grouped together. As mentioned earlier, the simultaneous incorporation of phenotypic and metabolomic data resulted in overconstraining the solution space (e.g. drug response prediction), especially for PRIME^c^. Nonetheless, TRFBA^c^ was positively influenced by cell-specific constraints, presumably due to its more flexible approach to adjust the bounds of reactions. Interestingly, pFBA^c^ performed better than several context-specific algorithms in comparison benchmarks, suggesting the pivotal role of employing metabolomic data in deciphering underlying mechanisms of human diseases [52, 58, 59]. Methods in this group resulted in satisfactory predictions over comparison, and fair performance in consistency benchmarks. Finally, group 3 included FASTCORE, INIT, iMAT and FASTCORMICS. FASTCORE and FASTCORMICS iteratively solve a set of LP problems that maximize the number of core reactions, while minimizing the inclusion of non-core reactions. Moreover, iMAT and INIT maximize the consistency between experimental data and *in silico* predictions, and therefore find a trade-off between inclusion and exclusion of highly- and lowly-expressed reactions, respectively. [23]. Although the mathematical frameworks of the algorithms in this group are different, they performed similarly over different benchmarks. Notably, FASTCORMICS and iMAT, and FASTCORE and INIT grouped together in smaller sub-clusters. The low expression cutoff in iMAT resulted in inclusion of biomass function in a number of models similar to that of the FASTCORMICS, which explain the capability of both methods to perform some comparison tests; however, FASTCORE and INIT failed to generate functional GEMs in the criteria used here. Nevertheless, the methods in this category showed a moderate to strong performance in consistency tests.

**Fig 12:**
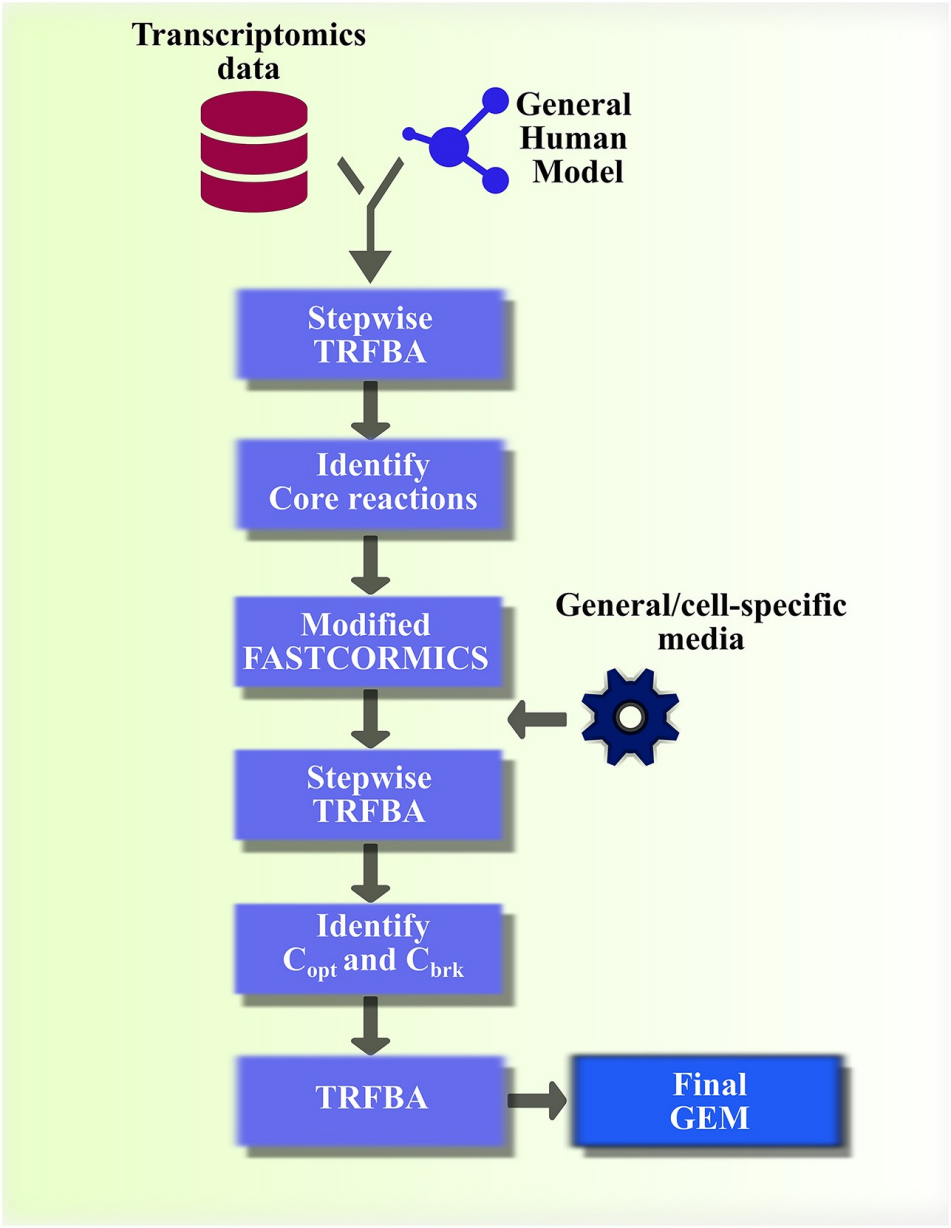
TRFBA-CORE workflow. TRFBA-CORE employs the stepwise version of TRFBA to identify a set of growth-associated reactions, build cell-specific models using modified FASTCORMICS, and generate tuned cell-specific GEMs.

Although there is no “perfect” algorithm which can satisfactorily pass all the benchmarks, FASTCORMICS, TRFBA and PRIME performed relatively better in consistency and comparison tests, respectively. Hence, by taking a benchmark-driven approach, we focused our attention on designing a context-specific algorithm by exploiting these algorithms. It is worth mentioning that previous efforts were mainly focused on general benchmarking of metabolic modeling algorithms [21–24]. Thus, designing context-specific algorithms by adapting, customizing and modifying advantageous features of powerful algorithms for cancer seems a promising avenue to explore. In the following, we introduced TRFBA-CORE, and explained its developmental stages based on modified characteristics of the afore-mentioned methods. As shown in Fig 12, TRFBA-CORE comprises of two main steps: 1- identifying growth-correlated reactions by stepwise TRFBA, and generating GEMs using modified FASTCORMICS (S3 Text), and 2- identifying correlation C (C_corr_), and optimal C (C_opt_) in case of available phenotypic data (e.g. growth rates).

#### Growth-correlated reactions

TRFBA employs a constant parameter, C, to convert expression levels to upper bounds of gene associated reactions. This parameter is determined from a sensitivity analysis on growth prediction error [18].Therefore, C is dependent on input general model, expression profiles, and most importantly, a priori knowledge of experimental growth rate data. In general, the use of experimental data to determine the maximum possible flux values through the network reactions has been previously explored in E-flux and PRIME algorithms [10, 60]; however, the way in which these algorithms deal with constraining the upper bounds of network reactions highly influences the resulting phenotypic behavior [10, 18].

Further examination of TRFBA revealed a strong positive monotonic relationship between C and predicted growth (S1 Fig), implying that a stepwise change in C leads to a gradual variation of predicted growth. From this perspective, varying C from the point it begins to affect the objective function (denoted as C_brk_) to 0 (full constraint), gradually narrows down the flux intervals and solution space (S1 Fig). Hence, there exists a set of reactions in the metabolic network for which the expression of their enzyme-coding genes (or reaction expression) varies monotonically with the flux through the biomass reaction. Based on this observation, we defined “growth-correlated reactions” as the set of reactions for which there is a strong correlation (using the Spearman correlation coefficients, corrected for FDR) between their flux values and predicted growth rates during a stepwise change in C. It should be noted that, the number of points used to discretize [0, C_brk_] interval may affect the size of resulting growth-correlated reactions. We therefore, determined different sets of growth-correlated reactions using different discretization intervals, and calculated the Jaccard scores for the resulting sets. Our analysis showed a strong similarity between the generated sets of growth-correlated reactions (S2 Fig). Hence, we selected the minimum step-size (500), above which no significant improvement in GEMs performance was achieved.

As shown here and previously, PRIME utilizes experimental cell growth rate data to identify a set of growth-associated reactions to be constrained in the output GEM [10]. In addition, it has recently been shown that the decision on gene expression threshold for identifying core reactions or stratifying them into active/inactive categories, profoundly affects the resulting GEM structure in algorithms adopting such approaches [23].Thus, regardless of whether the algorithms constrain the upper bound of reactions (such as PRIME and TRFBA) or categorize expression data to define a set of core reactions (such as FASTCORE family and GIMME), they depend on either *a priori* knowledge of experimental growth rates or a proper expression threshold to define a set of meaningful core reactions. Here, growth-correlated reactions do not hinge on the optimized C, and consequently on experimental growth rates. We next used a modified version of FASTCORMICS (S3 Text) to feed with this set of growth-correlated reactions to generate tailored GEMs, which were expected to increase the context-specificity of resulting networks (Fig 12).

#### Definition of C_corr_ and C_opt_ parameters

While the GEMs generated above were functional, and contained the set of growth-correlated reactions, the decision on C independent of experimental data, is however challenging. Based on our findings from the benchmarking section, we hypothesized that C is associated with the level of integration between expression data and metabolic network, which is fine-tuned when cell-specific media or other phenotypic data are applied. To assess this hypothesis, we tried to maximize the integration level by minimizing the Euclidean distance between flux rates and expression levels. In the original TRFBA, the linear inequality constraints describing the relationship between reaction fluxes and expression levels can be converted to equality constraint by adding variables to left-hand side of the equation:

$$\sum_{i\in R}v_{i}+\alpha_{j}=C\times E_{j}$$ (3)

where *R* corresponds to the set of reactions associated with gene *j*. Therefore, minimizing the above-mentioned Euclidean distance can be replaced by minimizing the Euclidean norm of introduced variables (α). Thus, the resulting quadratic programming (QP) problem can be written as:

$$\begin{array}{l} \text{min}\begin{array}{cc} & \sum_{k\in [0,C_{brk}]}^{}\alpha_{k}^{2} \end{array}\\ s.t.\begin{array}{cc} & \begin{array}{l} S.v=b\\ v_{lb}\leq v\leq v_{ub}\\ \sum_{i\in R}v_{i}+\alpha_{j}=C\times E_{j} \end{array} \end{array} \end{array}$$ (4)

The solution to the above QP problem using stepwise TRFBA will result in a matrix of flux distributions with rows corresponding to the reactions in the reconstructed GEMs and columns corresponding to C iterations. To measure the level of consistency between expression levels and predicted fluxes, we examined the number of variables (***N***_***α***_) at each iteration, that falls below a threshold (here, 1e-6). We observed that, the points (C_corr_) corresponding to the first sudden change (detected using MATLAB built-in function *FindChangePts*) in the ***N***_***α***_, resulted in significant correlation between predicted and measured growth rates for both general and cell-specific media (Table 3). Interestingly, we observed similar results (Table 3) when we used Recon 2 model [61]. The rationale behind this approach is that, a sudden increase in ***N***_***α***_ may represent a change in the network flux state, and corresponds to higher consistency between predicted fluxes and expression profiles. Therefore, the C_corr_ is the maximum point at which there is a shift in the flux consistency of the network.

**Table 3:** Spearman correlation coefficients between predicted and measured growth rates for C_corr_ and C_opt_.

Network	C	Constraints			
		General		Cell-specific	
		R	p-value	R	p-value
Recon 1	C_corr_	0.28	0.028	0.49	5.9e-5
	C_opt_	> 0.99	< 4.1e-125	1	0
Recon 2	C_corr_	0.27	0.03	0.37	0.003
	C_opt_	> 0.99	< 1.9e-95	> 0.99	< 3.6e-92

It is of interest to note that, when observed growth rates of cancerous cell lines are available, TRFBA-CORE can benefit from optimal cell-specific C values (C_opt_). In this case, C_opt_ for each cell-line is easily approximated by a linear function of measured growth rates, and eliminates the need for sensitivity analysis as in the original TRFBA implementation [18]:

C=optCbrkGmax×Gmeasured (5)

where *G*_*max*_ and *G*_*measured*_ are maximum predicted growth and measured growth rates, respectively. As expected, the predicted growth rates using C_opt_ values obtained from the above linear function showed a strong correlation to measured growth rates (Table 3).

As the final step of TRFBA-CORE, calculated values for C_corr_ and C_opt_ were used (a total of 4 different variations of TRFBA-CORE with general/cell-specific media, and with C_corr_/C_opt_) to generate functional cell-specific models, which were then examined for their performance in benchmarks used here.

#### Comparison benchmarks

TRFBA-CORE GEMs were evaluated for their predictive performance against growth rates (S3 Fig), metabolites uptake/secretion rates (S4 Fig) and drug response (S5 Fig). Since, TRFBA-CORE estimates C_opt_ more accurately, it is not surprising that it predicted the measured growth rates significantly better than other algorithms (Fig 1). Interestingly, contrary to other methods, TRFBA-CORE was able to predict a number of metabolites by taking into account only transcriptomic data (S4 Fig). A further improvement in prediction range was achieved by using cell-specific medium and C_copt_. Moreover, TRFBA-CORE performed significantly better than other methods in predicting drug responses; however, similar to PRIME^c^, the GEMs constrained with cell-specific media failed to achieve physiologically relevant results. Furthermore, TRFBA-CORE showed a similar performance to the original TRFBA in predicting the cell line-specific essential genes (S6 Fig), and a fair performance in enrichment analysis of the oncogenes (OG), tumor suppressors (TS) and loss-of-function mutations (LOFs) (S7 Fig).

#### Consistency benchmarks

**Fig 13:**
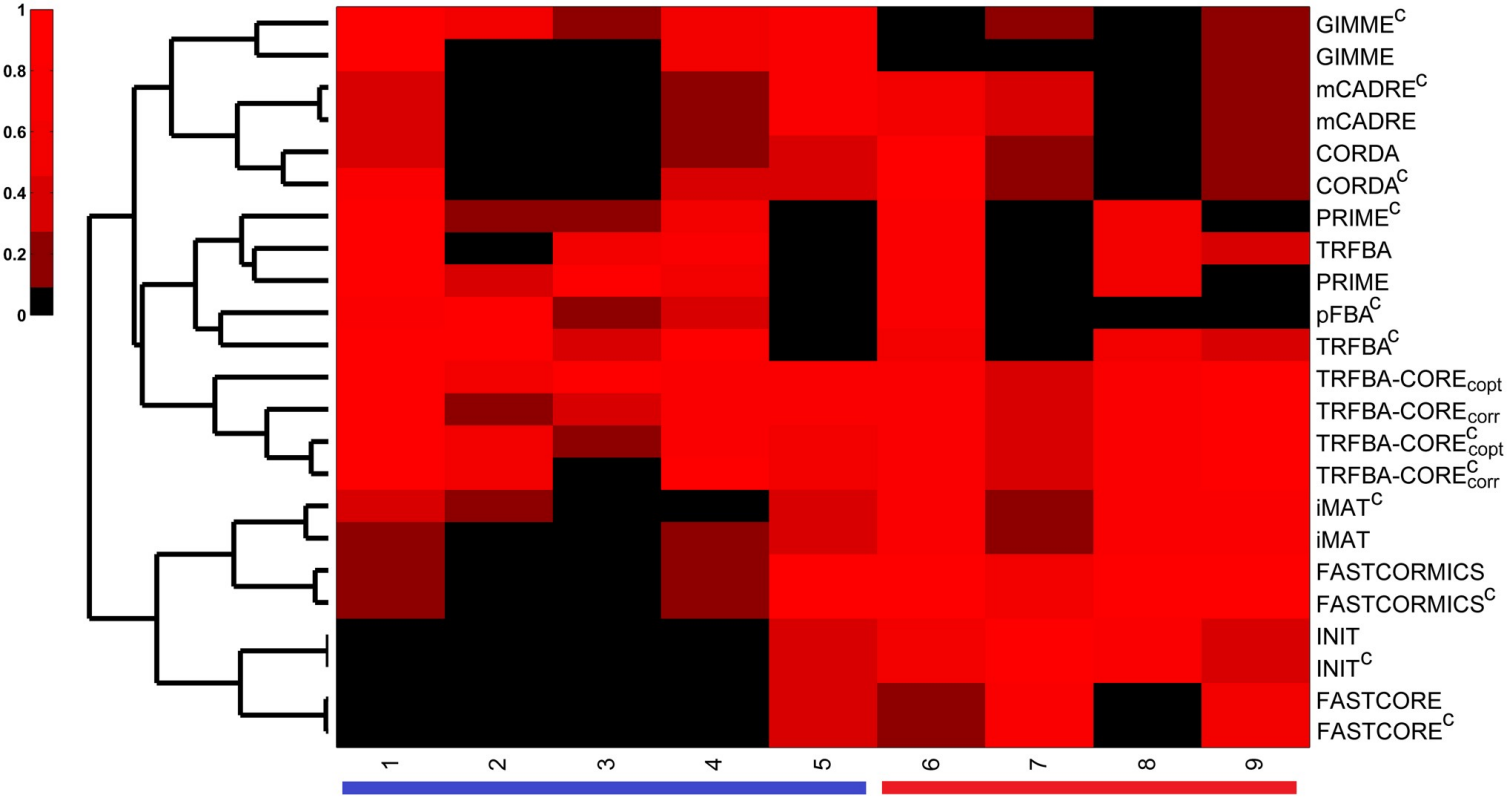
Hierarchical clustering of TRFBA-CORE performance scores. Hierarchical clustering (Euclidean distance) of the scores TRFBA-CORE received in different benchmarks. Despite being a GEM extraction approach, TRFBA-CORE was clustered with algorithms that do not trim the input model. TRFBA-CORE scores were generally higher in comparison benchmarks. Numbers in column correspond to comparison (blue color) or consistency (red color) benchmarks: 1-growth rate, 2- metabolite uptake/secretion rates, 3- drug response, 4- essential genes, 5- enrichment of OG/TS/LOFs, 6- fraction of blocked reactions, 7- resolution power, 8- robustness to missing data, and 9- robustness to noise. Colorbar indicates normalized performance scores.

TRFBA-CORE contained a lower fraction of blocked reactions compared to TRFBA, which can be attributed to the use of FASTCORMICS in the reconstruction process (S8 Fig). Moreover, while the resolution power of TRFBA-CORE was comparatively better than most competitors (S9 Fig), it failed to surpass FASTCORE, FASTCORMICS or INIT, which seems to be due the focus of TRFBA-CORE on growth-correlated reactions [62]. Nevertheless, while the growth rate predictions of TRFBA-CORE were sensitive to input missing reactions (S10 Fig), it showed a better capability in recovering the missing reactions compared to most of the methods (hypergeometric p-value < 5e-14). It should be noted that, since input reactions for TRFBA-CORE are growth-correlated (and identified based on different flux states), their removal from the input core reactions markedly affect the growth predictions. However when a fraction of input gene expression data were missing (similar to the approach we used for TRFBA and PRIME), TRFBA-CORE exhibited a significantly increased robustness to growth rate predictions compared to TRFBA. Furthermore, the growth robustness of the GEMs generated by TRFBA-CORE was similar to the trend observed for iMAT and FASTCORMICS (S11 Fig); however, in terms of the similarity level (S12 Fig), its robustness to the noise in input gene expression data was weak (similar to INIT). We next used our scoring scheme to cluster the performance scores of TRFBA-CORE (Fig 13).

Surprisingly, while TRFBA-CORE is a subnetwork extraction method, it was grouped with the methods that retain the general input model. GEMs generated with 4 variations of TRFBA-CORE showed a satisfactory performance over both comparison and consistency benchmarks. It is of interest to note that the inclusion of growth-correlated reactions improved the functional ability of FASTCROMICS and consistency performance of TRFBA, the two ancestors of TRFBA-CORE. However, it is of importance to note that, TRFBA-CORE requires further improvements in terms of its ability to generate cell/tissue-specific models with higher robustness of identified core reactions (e.g. growth-correlated reactions) to the step-wise conversion of gene expression data to reaction upper bounds. Yet, the current benchmark-driven approach can provide guidelines for the development of more advanced reconstruction methods with better capability to recapitulate various features of cancer metabolism.

### Conclusion

Here, we employed a variety of structural and functional benchmarks to examine the predictive performance of different algorithms developed to study cancer metabolism. These benchmarks reflect quantitative rather than mere qualitative aspects of algorithms studied here. We compared the performance of several algorithms, classified them based on their performance, and found inconsistencies in the predictive capability of these methods. Moreover, we showed that employing physiologically meaningful media using metabolomics (or possibly fluxomics) can greatly improve the functional performance of the computational methods, pointing out the need for more attention to medium uptake rates. Finally, we developed a computational approach based on results obtained from benchmarks utilizing algorithmic features of methods with highest predictive performance across different tests. The benchmark-driven approach developed here outperformed several methods in a number of tests. TRFBA-CORE, unlike its ancestors, does not rely on prior knowledge of phenotypic data (e.g. growth rates), and only takes advantages of expression data. However, inclusion of high-quality phenotypic and omics data will improve the predictive power of current method, as we found in the case of TRFBA. TRFBA-CORE will be further explored in our future work to find potential novel drug targets for cancer treatment.

## Supporting information

S1 Text Parameter optimization. The sensitivity analysis for GIMME, iMAT, CORDA and TRFBA. Adjustable parameters of each algorithm were selected based on their performance in growth rate prediction.
(PDF)

S2 Text Scoring scheme for performance assessment of methods. All methods studied here were assigned a numerical score based on their performance across different benchmarks.
(PDF)

S3 Text Modified FASTCORMICS. FASTCORMICS was assessed with regard to the assumptions made by the original implementation to improve the capability of the method to generate functional GEMs.
(PDF)

S1 Fig Schematic of existing monotonic relationship between C and predicted growth. The breaking point (C_brk_) denotes the C value at which further reduction in C affects the predicted growth rate.
(TIF)

S2 Fig Pairwise similarity indices for sets of identified growth-correlated reactions at different step-sizes (50–2000). The indices are shown as the mean value across all cell lines in NCI-60 panel.
(TIF)

S3 Fig Growth rate prediction for TRFBA-CORE. Distribution of relative error for prediction of growth rates for 4 variations of TRFBA-CORE (with general/cell-specific media, and C_opt_/C_corr_). Each box-plot shows the distribution of error across all cell lines in NCI-60 panel.
(TIF)

S4 Fig Uptake/secretion rates prediction for TRFBA-CORE. Spearman correlation between measured and predicted uptake/secretion flux rates of metabolites for (A) TRFBA-CORE_corr_, (B) TRFBA-CORE_opt_, (C) TRFBA-CORE^c^_corr_, and (D) TRFBA-CORE^c^_opt_. Represented p-values were adjusted for False discovery rate (α = 0.05).
(TIF)

S5 Fig Drug response predictions for TRFBA-CORE. Heatmap of significant Spearman correlations between simulated and experimental drug response data for 4 variations of TRFBA-CORE (with general/cell-specific media, and C_opt_/C_corr_). The Spearman coefficients for each drug have been shown on the figure. Superscripts indicate drug response data taken from (1) Holbeck et al [38], (2) Garnett et al [39] and (3) Yang et al [40].
(TIF)

S6 Fig Prediction of general cancer essential genes for TRFBA-CORE. Heatmap of enrichment p-values for predicted cell-line specific essential genes for 4 variations of TRFBA-CORE (with general/cell-specific media, and C_opt_/C_corr_). The numbers indicate -log10 enrichment p-values.
(TIF)

S7 Fig Prediction of OG, TS and LOF mutations for TRFBA-CORE. Mean enrichment of predicted OGs and and LOFs with experimental data. The error bar indicates the standard deviation across GEMs generated with general and cell-specific media. Hypergeometric p-values are shown above the figure. Model fraction represents the fraction of generated GEMs with significant p-values (<0.05).
(TIF)

S8 Fig Network connectivity analysis for TRFBA-CORE. The presence of blocked reactions were assessed in both constrained and unconstrained states for 4 variations of TRFBA-CORE (with general/cell-specific media, and C_opt_/C_corr_). Data shown as mean fraction of existing blocked reaction across all generated GEMs, and error bars represent the standard deviation.
(TIF)

S9 Fig Similarity levels of TRFBA-CORE GEMs between different tumors. Average Jaccard similarity index computed for GEMs built by (A) TRFBA-CORE and (B) TRFBA-CORE^c^. Each square represents the average pairwise Jaccard value for each cancer type in the NCI-60 panel.
(TIF)

S10 Fig Normalized growth prediction of TRFBA-CORE GEMs generated using data from repeated 5-fold cross-validation. Model count represents the GEMs generated by incomplete growth-correlated reactions in the input.
(TIF)

S11 Fig Normalized growth prediction of TRFBA-CORE GEMs generated using noisy expression data. The x-axis shows the spearman correlation coefficient between each set of noisy data and original expression profile ranging from 1 (original) to R < 0.004 (random).
(TIF)

S12 Fig Similarity levels of TRFBA-CORE GEMs generated with different sets of noisy expression data. (TIF)

S1 File Graphical User Interface (GUI) of benchmark panel. MATLAB GUI application for benchmark tests, along with all experimental datasets used here.
(7Z) Click here for additional data file.

S2 File MATLAB scripts for TRFBA-CORE. (7Z)
