# Supplementary material for: A benchmark-driven approach to reconstruct metabolic networks for studying cancer metabolism
Source: PLoS Comput Biol. 2019 Apr 22;15(4):e1006936. doi: 10.1371/journal.pcbi.1006936 (PMC6497301; doi:10.1371/journal.pcbi.1006936)
Supplement: S1 Text — The sensitivity analysis for GIMME, iMAT, CORDA and TRFBA. Adjustable parameters of each algorithm were selected based on their performance in growth rate prediction. (PDF) [file pcbi.1006936.s001.pdf]

S1 Text. **A benchmark-driven approach to reconstruct metabolic networks for studying cancer metabolism**

Oveis Jamialahmadi, Sameereh Hashemi-Najafabadi, Ehsan Motamedian, Stefano Romeo, Fatemeh Bagheri

- **Parameter optimization**

GIMME. GEMs were generated by simultaneously varying the fraction of objective function and gene expression threshold linearly from 10^{-6} to 1 (11 points), and between 1st and 99th percentile of the input expression profile (21 points), respectively. Figure A shows the simultaneous effect of 2 adjustable parameters of GIMME in terms of growth rate prediction (Figure A (a)) and the number of non-empty GEMs (Figure A (b)). While the sensitivity of GIMME to expression threshold was relatively low (except at moderate objective fractions), the decision on the fraction of objective function (i.e. growth) greatly affected the growth predictions, which was mainly due to the large difference between measured and predicted growth rates for cancer cell lines. The parameters corresponding to the lower relative error and higher model count (i.e. number of GEMs) were selected for all simulations (i.e. the fraction of objective function = 0.1 of maximum growth rate and expression threshold = 6th percentile).

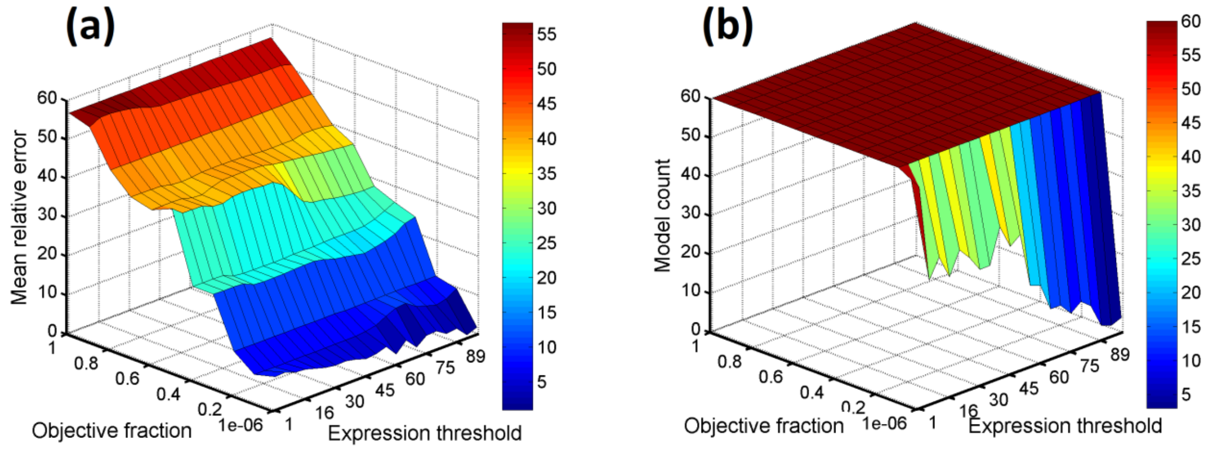

Figure A. Parameter optimization for GIMME. More than 13500 GEMs were generated by simultaneously varying the fraction of objective function and gene expression threshold. The 2 adjustable parameters were optimized based on the performance in growth rate predictions (a), and number of functional GEMs (b).

iMAT. GEMs were generated by simultaneously varying 3 parameters of the algorithm, namely, flux activation threshold (ε), low and high expression thresholds were selected based on sensitivity analysis. More than 14500 GEMs were generated by simultaneously varying the flux activation threshold (ε) from 10^{-3} to 10 (log-scale, 5 points), low expression threshold from 1st to 75th (7 points) percentile, and high expression threshold from 25th to 99th percentile (7 points). Figure B shows the simultaneous effect of 3 adjustable parameters of iMAT in terms of growth rate prediction and the number of non-empty (functional) GEMs. As depicted, the number of functional GEMs was profoundly affected by increasing ε (Figure B (f, h and j)). Moreover, at lower ε values, lower expression thresholds resulted in better growth rate predictions and higher number of functional GEMs. Best results were achieved when high expression threshold was set to 25th percentile (Figure B (a and b)). Therefore, moderately expressed genes in the default parameters (i.e. low threshold= 25th, and high threshold = 75th percentile) were considered as highly expressed genes [1].

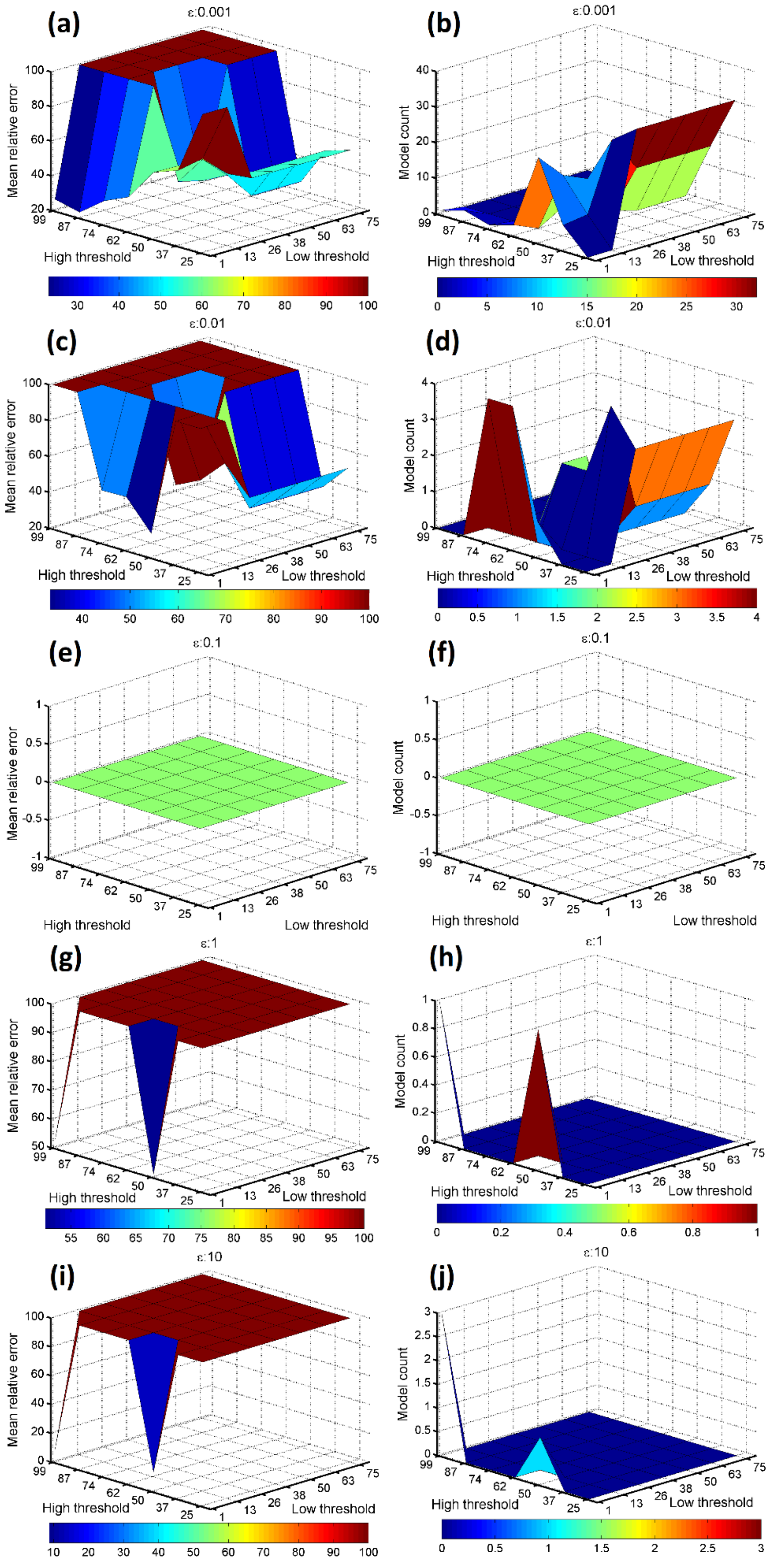

(a)
ε:0.001
Mean relative error
High threshold
Low threshold
(b)
ε:0.001
Model count
High threshold
Low threshold
(c)
ε:0.01
Mean relative error
High threshold
Low threshold
(d)
ε:0.01
Model count
High threshold
Low threshold
(e)
ε:0.1
Mean relative error
High threshold
Low threshold
(f)
ε:0.1
Model count
High threshold
Low threshold
(g)
ε:1
Mean relative error
High threshold
Low threshold
(h)
ε:1
Model count
High threshold
Low threshold
(i)
ε:10
Mean relative error
High threshold
Low threshold
(j)
ε:10
Model count
High threshold
Low threshold

Figure B. Parameter optimization for iMAT. More than 14500 GEMs were generated by simultaneously varying the flux activation threshold (ε), low and high expression thresholds. The 3 adjustable parameters were optimized based on the performance in growth rate predictions (a, c, e, g and i), and number of functional GEMs (b, d, f, h and j).

CORDA. GEMs were generated by linearly varying the constraint value for defining the reaction dependency from 1% to 99% of maximal flux rate (20 points). As shown in Figure C, the decision on this parameter did not affect the generated GEMs. Therefore, the constraint was set to 10% of maximal flux rate.

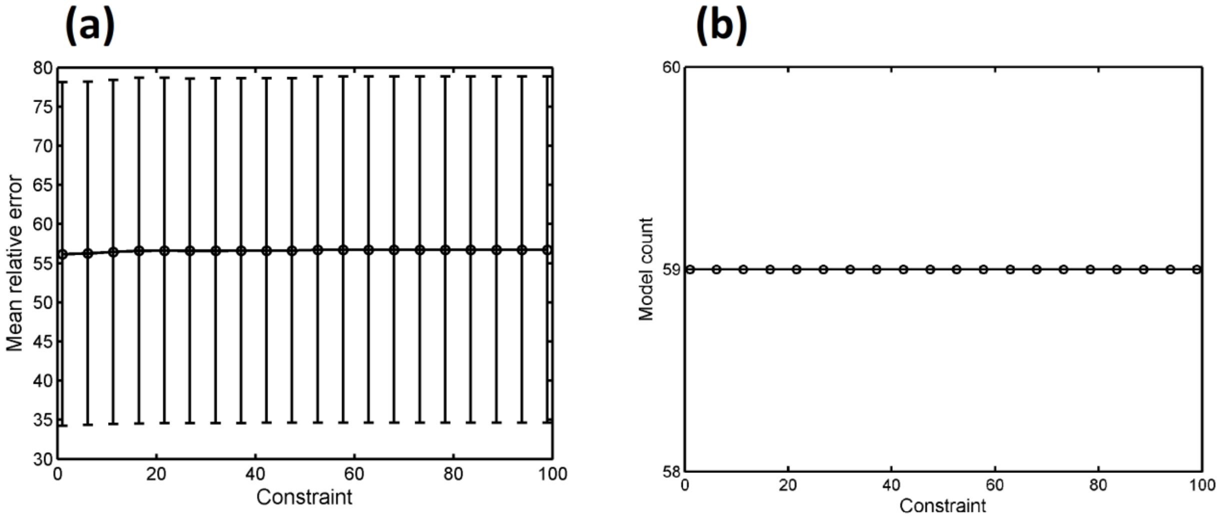

Figure C. Parameter optimization for CORDA. A total of 1200 GEMs were generated by linearly varying the constraint parameter from 1% to 99% of maximum flux rate. The parameter did not exhibit any evident effect on predicted growth rates (represented as mean relative error ± standard deviation) (a) or the number of functional GEMs (b).

TRFBA. The constant parameter C in TRFBA was calculated according to the original publication [2]. To determine the range at which TRFBA shows a non-monotonic dependence on growth rate, C was varied between several extreme points (from 0 to 1). Next, the C was varied linearly from

0.03 to 0.05 (15 points) and the mean relative error was calculated at each C. The C corresponding to the minimum relative error (C = 0.0405) was selected for all simulations (Figure. D).

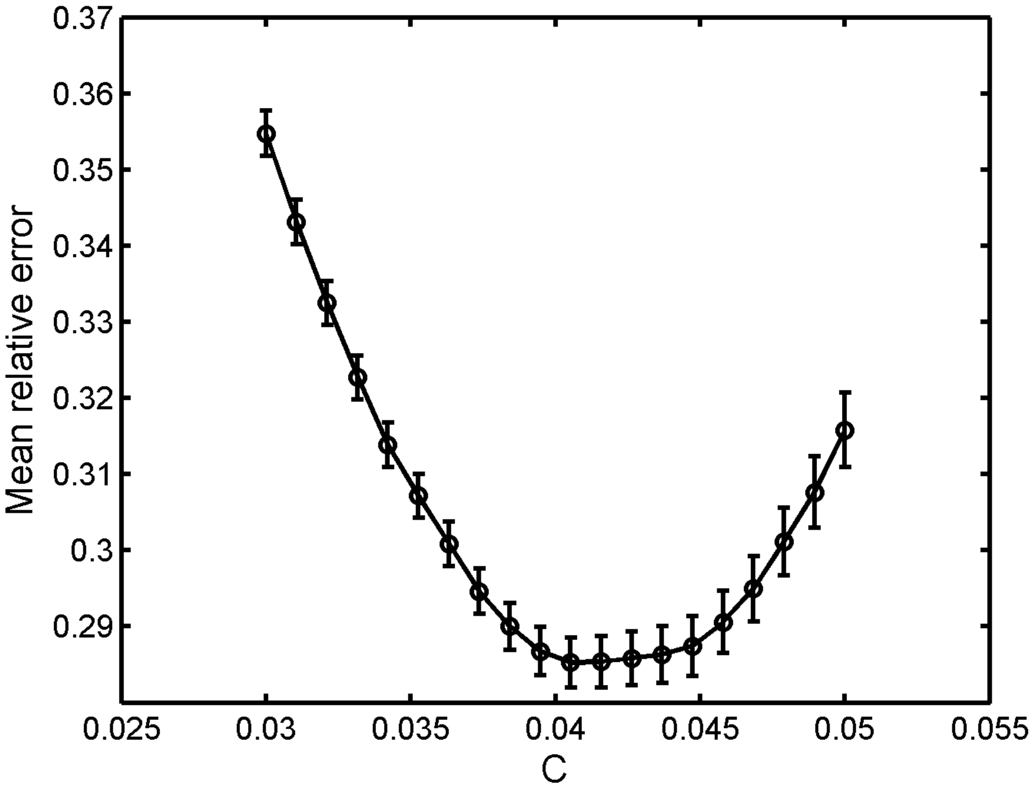

Figure D. Parameter optimization for TRFBA. GEMs were generated by linearly varying the parameter C between 0.03 and 0.05. Data shown as mean relative error ± standard error.

References

1. Shlomi T, Cabili MN, Herrgård MJ, Palsson BØ, Ruppin E. Network-based prediction of human tissue-specific metabolism. Nat Biotechnol. 2008; 26(9):1003-1010.
2. Motamedian E, Mohammadi M, Shojaosadati SA, Heydari M. TRFBA: an algorithm to integrate genome-scale metabolic and transcriptional regulatory networks with incorporation of expression data. Bioinformatics. 2017; 33(7):1057-1063.
