# Supplementary material for: A benchmark-driven approach to reconstruct metabolic networks for studying cancer metabolism
Source: PLoS Comput Biol. 2019 Apr 22;15(4):e1006936. doi: 10.1371/journal.pcbi.1006936 (PMC6497301; doi:10.1371/journal.pcbi.1006936)
Supplement: S2 Text — All methods studied here were assigned a numerical score based on their performance across different benchmarks. (PDF) [file pcbi.1006936.s002.pdf]

S2 Text. **A benchmark-driven approach to reconstruct metabolic networks for studying cancer metabolism**

Oveis Jamialahmadi, Sameereh Hashemi-Najafabadi, Ehsan Motamedian, Stefano Romeo, Fatemeh Bagheri

Scoring scheme for the performance assessment of methods. The performance of each method across comparison and consistency benchmarks was converted to a quantitative score as described in the following paragraphs.

1- **Growth rate predictions.** Each method received a score based on the fraction of its functional GEMs (i.e. capable of predicting non-zero growth rates), and the mean relative error (lower is better) across all generated GEMs:

$$\boldsymbol{S_{growth}} = (\mathbf{100} - \boldsymbol{\varepsilon_{mean}}) \times \boldsymbol{f} \qquad \mathbf{(1)}$$

where $\boldsymbol{\varepsilon_{mean}}$ is the mean relative error, and $\boldsymbol{f}$ is the fraction of functional GEMs. The resulting vector of performance scores was normalized to the maximum score (Table A).

Table A. Performance scores of growth rate predictions. Normalized score$^{\text{TRFBA-CORE}}$ was calculated by taking into account the performance scores of TRFBA-CORE.

Method	ε_{mean}	f	Score	Normalized score	Normalized score$^{\text{TRFBA-CORE}}$
**CORDA**	56.36	1	43.64	0.44	0.44
**CORDA^c**	20.31	1	79.69	0.81	0.80
**FASTCORE**	0	0	0	0	0
**FASTCORE^c**	0	0	0	0	0
**FASTCORMICS**	4.08	0.25	24.39	0.25	0.24
**FASTCORMICS^c**	1.30	0.17	16.73	0.17	0.17
**GIMME**	7.26	1	92.74	0.94	0.93
**GIMME^c**	2.62	1	97.38	0.99	0.98
**INIT**	0	0	0	0	0
**INIT^c**	0	0	0	0	0
**PRIME**	7.16	1	92.84	0.94	0.93
**PRIME^c**	3	1	97	0.98	0.97
**TRFBA**	3.97	1	96.03	0.97	0.96
**TRFBA^c**	1.22	1	98.78	1	0.99
**iMAT**	54.93	0.53	24.04	0.24	0.24
**iMAT^c**	19.15	0.37	29.64	0.30	0.30
**mCADRE**	1.87	0.41	39.92	0.40	0.40
**mCADRE^c**	0.73	0.39	38.70	0.39	0.39
**pFBA^c**	19.01	1	80.99	0.82	0.81
**TRFBA-CORE$_{corr}$**	6.94	1	93.06	-	0.93
**TRFBA-CORE$^c_{corr}$**	1.51	1	98.49	-	0.99
**TRFBA-CORE$_{copt}$**	0.67	1	99.33	-	1
**TRFBA-CORE$^c_{copt}$**	0.38	1	99.62	-	1

2- **Prediction of metabolite uptake/secretion rates.** Each method received a score based on the number of predicted metabolites and mean Spearman correlation coefficient of all predicted metabolites:

$$\boldsymbol{S_{mets} = R_{mean} \times N_{mets}} \quad \textbf{(2)}$$

where $\boldsymbol{R_{mean}}$is the mean Spearman coefficient for the predicted metabolites, and $\boldsymbol{N_{mets}}$is the number of significantly predicted metabolites. The resulting vector of performance scores was normalized to the maximum score (Table B).

Table B. Performance scores of metabolite uptake/secretion rates. Normalized score$^{TRFBA\text{-}CORE}$ was calculated by taking into account the performance scores of TRFBA-CORE.

Method	N_{mets}	R_{mean}	Score	Normalized score	Normalized score$^{TRFBA\text{-}CORE}$
**CORDA**	0	0	0	0	0
**CORDA^c**	0	0	0	0	0
**FASTCORE**	0	0	0	0	0
**FASTCORE^c**	0	0	0	0	0
**FASTCORMICS**	0	0	0	0	0
**FASTCORMICS^c**	0	0	0	0	0
**GIMME**	0	0	0	0	0
**GIMME^c**	11	0.71	7.82	0.62	0.62
**INIT**	0	0	0	0	0
**INIT^c**	0	0	0	0	0
**PRIME**	13	0.41	5.34	0.42	0.42
**PRIME^c**	5	0.61	3.04	0.24	0.24
**TRFBA**	0	0	0	0	0
**TRFBA^c**	20	0.63	12.63	1	1
**iMAT**	0	0	0	0	0
**iMAT^c**	5	0.45	2.23	0.18	0.18
**mCADRE**	0	0	0	0	0
**mCADRE^c**	0	0	0	0	0
**pFBA^c**	15	0.72	10.75	0.85	0.85
**TRFBA-CORE$_{corr}$**	7	0.40	2.78	-	0.22
**TRFBA-CORE$^c_{corr}$**	14	0.46	6.48	-	0.51
**TRFBA-CORE$_{copt}$**	11	0.53	5.83	-	0.46
**TRFBA-CORE$^c_{copt}$**	14	0.55	7.68	-	0.61

3- **Drug response predictions.** Each method received a score based on the number of predicted drugs and mean Spearman correlation coefficient of all predicted drug responses:

$$\boldsymbol{S_{drug} = R_{mean} \times N_{drug}} \tag{3}$$

where $\boldsymbol{R_{mean}}$is the mean Spearman coefficient for the predicted drug responses, and $\boldsymbol{N_{drug}}$is the number of significantly predicted drug responses. The resulting vector of performance scores was normalized to the maximum score (Table C).

Table C. Performance scores of drug response predictions. Normalized score$^{TRFBA\text{-}CORE}$ was calculated by taking into account the performance scores of TRFBA-CORE.

Method	N_{drug}	R_{mean}	Score	Normalized score	Normalized score$^{TRFBA\text{-}CORE}$
**CORDA**	1	0.26	0.26	0.08	0.06
**CORDA^{c}**	0	0	0	0	0
**FASTCORE**	1	0.30	0.30	0.09	0.08
**FASTCORE^{c}**	1	0.30	0.30	0.09	0.08
**FASTCORMICS**	0	0	0	0	0
**FASTCORMICS^{c}**	0	0	0	0	0
**GIMME**	0	0	0	0	0
**GIMME^{c}**	2	0.30	0.59	0.18	0.15
**INIT**	0	0	0	0	0
**INIT^{c}**	0	0	0	0	0
**PRIME**	8	0.42	3.37	1	0.84
**PRIME^{c}**	1	0.37	0.37	0.11	0.09
**TRFBA**	6	0.37	2.22	0.66	0.55
**TRFBA^{c}**	5	0.31	1.56	0.46	0.39
**iMAT**	0	0	0	0	0
**iMAT^{c}**	1	0.26	0.26	0.08	0.07
**mCADRE**	0	0	0	0	0
**mCADRE^{c}**	1	0.27	0.27	0.08	0.07
**pFBA^{c}**	2	0.31	0.62	0.18	0.15
**TRFBA-CORE$_{corr}$**	5	0.34	1.72	-	0.43
**TRFBA-CORE$^{c}_{corr}$**	0	0	0	-	0
**TRFBA-CORE$_{copt}$**	8	0.50	4	-	1
**TRFBA-CORE$^{c}_{copt}$**	1	0.46	0.46	-	0.11

4- **Prediction of cell line-specific essential genes.** Each method received a score based on the mean enrichment p-values (log-transformed) and the fraction of significant cell lines (of 22):

$$S_{essentiality} = -\log_{10} P \times f \quad (4)$$

where $-\log_{10} P$ denotes the mean enrichment p-values (log-transformed), and f is the fraction of GEMs (of 22) with significant predictions. The resulting vector of performance scores was normalized to the maximum score (Table D).

Table D. Performance scores of gene essentiality predictions. Normalized score$^{\text{TRFBA-CORE}}$ was calculated by taking into account the performance scores of TRFBA-CORE.

Method	f	$-\log_{10} P$	Score	Normalized score	Normalized score$^{\text{TRFBA-CORE}}$
**CORDA**	0.64	2.22	1.41	0.20	0.20
**CORDA^c**	1	2.57	2.57	0.36	0.36
**FASTCORE**	0	0	0	0	0
**FASTCORE^c**	0	0	0	0	0
**FASTCORMICS**	0.27	3.94	1.08	0.15	0.15
**FASTCORMICS^c**	0.23	4.21	0.96	0.14	0.14
**GIMME**	1	3.69	3.69	0.52	0.52
**GIMME^c**	1	3.73	3.73	0.53	0.53
**INIT**	0	0	0	0	0
**INIT^c**	0	0	0	0	0
**PRIME**	1	3.86	3.86	0.54	0.54
**PRIME^c**	0.95	3.61	3.45	0.49	0.49
**TRFBA**	1	4.84	4.84	0.68	0.68
**TRFBA^c**	1	7.09	7.09	1	1
**iMAT**	0.36	2.32	0.84	0.12	0.12
**iMAT^c**	0.27	2.14	0.58	0.08	0.08
**mCADRE**	0.41	4.72	1.93	0.27	0.27
**mCADRE^c**	0.41	4.31	1.76	0.25	0.25
**pFBA^c**	0.91	2.81	2.55	0.36	0.36
**TRFBA-CORE$_{corr}$**	1	4.94	4.94	-	0.70
**TRFBA-CORE$^c_{corr}$**	1	5.91	5.91	-	0.83
**TRFBA-CORE$_{copt}$**	1	5	5	-	0.71
**TRFBA-CORE$^c_{copt}$**	1	5.68	5.68	-	0.80

5- **Prediction of OG and TS/LOFs.** Each method received a score based on the mean fold enrichment for OG (higher is better) and for TS/LOFs (lower is better), and the fraction of GEMs with significant hypergeometric p-values:

$$S_{OG/TS} = \frac{E_{OG}}{E_{OG}^{max}} \times f_{OG} + \frac{E_{TS/LOF}^{max}}{E_{TS/LOF}} \times f_{TS/LOF} \quad (5)$$

where E_{OG} is the mean fold enrichment for OG, f_{OG} is the fraction of corresponding significant GEMs, $E_{TS/LOF}$ is the mean fold enrichment for TS/LOFs, and $f_{TS/LOF}$ is the fraction of corresponding significant GEMs. To be comparable, the vectors of fold enrichments were normalized to corresponding maximum values, so that the highest and lowest fold enrichment for OG and TS/LOF was equal to 1, respectively. The resulting vector of performance scores was then normalized to the maximum score (Table E).

Table E. Performance scores for predictions of OG and TS/LOFs. Normalized score$^{TRFBA\text{-}CORE}$ was calculated by taking into account the performance scores of TRFBA-CORE. NA indicates not applicable.

	OG			TS/LOF					
**Method**	f_{OG}	E_{OG}	$\frac{E_{OG}}{E_{OG}^{max}}$	$f_{TS/LOF}$	$E_{TS/LOF}$	$\frac{E_{TS/LOF}}{E_{TS/LOF}^{max}}$	**Score**	**Normalized score**	**Normalized score$^{TRFBA\text{-}CORE}$**
**CORDA**	0.25	1.07	0.67	0.11	0.94	1	0.28	0.30	0.30
**CORDA^c**	0.25	1.07	0.67	0.11	0.94	1	0.28	0.30	0.30
**FASTCORE**	0.31	1.21	0.76	0.05	0.85	1.11	0.29	0.31	0.31
**FASTCORE^c**	0.31	1.21	0.76	0.05	0.85	1.11	0.29	0.31	0.31
**FASTCORMICS**	0.63	1.43	0.90	0.29	0.75	1.25	0.93	1	1
**FASTCORMICS^c**	0.63	1.43	0.90	0.29	0.75	1.25	0.93	1	1
**GIMME**	1	1.06	0.67	0	0	0	0.67	0.73	0.73
**GIMME^c**	1	1.06	0.67	0	0	0	0.67	0.73	0.73
**INIT**	0.22	1.58	1	0.07	0.42	2.22	0.36	0.39	0.39
**INIT^c**	0.22	1.58	1	0.07	0.42	2.22	0.36	0.39	0.39
**PRIME**	NA	NA	NA	NA	NA	NA	NA	NA	NA
**PRIME^c**	NA	NA	NA	NA	NA	NA	NA	NA	NA
**TRFBA**	NA	NA	NA	NA	NA	NA	NA	NA	NA
**TRFBA^c**	NA	NA	NA	NA	NA	NA	NA	NA	NA
**iMAT**	0.53	1.08	0.68	0	0	0	0.36	0.39	0.39
**iMAT^c**	0.53	1.08	0.68	0	0	0	0.36	0.39	0.39
**mCADRE**	1	1.12	0.71	0.03	0.92	1.02	0.74	0.80	0.80
**mCADRE^c**	1	1.12	0.71	0.03	0.92	1.02	0.74	0.80	0.80
**pFBA^c**	NA	NA	NA	NA	NA	NA	NA	NA	NA
**TRFBA-CORE$_{corr}$**	0.43	1.19	0.75	0.25	0.82	1.14	0.61	-	0.66
**TRFBA-CORE$^c_{corr}$**	0.43	1.19	0.75	0.18	0.83	1.13	0.53	-	0.58
**TRFBA-CORE$_{copt}$**	0.43	1.19	0.75	0.25	0.82	1.14	0.61	-	0.66
**TRFBA-CORE$^c_{copt}$**	0.43	1.19	0.75	0.18	0.83	1.13	0.53	-	0.58

6- **Network connectivity.** Each method received a score based on the mean fraction of blocked reactions present in the generated GEMs in two constrained and unconstrained states:

$$\boldsymbol{S_{connectivity}} = \boldsymbol{f^{max}} - \boldsymbol{f} \quad \textbf{(6)}$$

where $\boldsymbol{f}$ is the mean fraction of blocked reactions in both constrained and unconstrained. Since, lower fractions of blocked reactions denote better performance, the distance to the maximum mean fraction was considered as the performance score. The resulting vector of performance scores was then normalized to the maximum score (Table F).

Table F. Performance scores for network connectivity. Normalized score$^{TRFBA\text{-}CORE}$ was calculated by taking into account the performance scores of TRFBA-CORE.

Method	*f*	Score	Normalized score	Normalized score$^{TRFBA\text{-}CORE}$
**CORDA**	0	0.49	1	1
**CORDA^{c}**	0	0.49	1	1
**FASTCORE**	0.38	0.10	0.21	0.21
**FASTCORE^{c}**	0.39	0.10	0.19	0.19
**FASTCORMICS**	0.04	0.45	0.91	0.91
**FASTCORMICS^{c}**	0.09	0.40	0.82	0.82
**GIMME**	0.49	0	0	0
**GIMME^{c}**	0.49	0	0	0
**INIT**	0.26	0.23	0.48	0.48
**INIT^{c}**	0.26	0.23	0.48	0.48
**PRIME**	0.11	0.38	0.77	0.77
**PRIME^{c}**	0.11	0.38	0.77	0.77
**TRFBA**	0.17	0.32	0.65	0.65
**TRFBA^{c}**	0.18	0.31	0.63	0.63
**iMAT**	0.12	0.37	0.75	0.75
**iMAT^{c}**	0.12	0.37	0.75	0.75
**mCADRE**	0.19	0.29	0.60	0.60
**mCADRE^{c}**	0.21	0.28	0.58	0.58
**pFBA^{c}**	0.11	0.38	0.77	0.77
**TRFBA-CORE$_{corr}$**	0.10	0.39	-	0.79
**TRFBA-CORE$^{c}_{corr}$**	0.11	0.38	-	0.77
**TRFBA-CORE$_{copt}$**	0.10	0.39	-	0.80
**TRFBA-CORE$^{c}_{copt}$**	0.11	0.38	-	0.78

7- **Similarity level.** Each method received a score based on the mean of differences between Jaccard index for each cancer type and others (i.e. the difference between the similarity of diagonal and others, for each column in Fig 7 of the main text). Therefore, the similarity level score for each cancer is calculated as:

$$S_{similarity}^{i} = \overline{J_{\iota} - J_{k=1,\ldots,9,k\neq\iota}} \quad \textbf{(7)}$$

where J_i is the Jaccard similarity index for cancer i. The overall score was then calculated as the mean score across 9 cancer types in the NCI-60 panel:

$$S_{similarity} = \frac{1}{9} \times \sum_{i=1}^{9} S_{similarity}^{i} \quad \textbf{(8)}$$

The resulting vector of performance scores was then normalized to the maximum score (Table G).

Table G. Performance scores of similarity levels. Normalized score$^{TRFBA\text{-}CORE}$ was calculated by taking into account the performance scores of TRFBA-CORE. NA indicates not applicable.

$S^{i}_{similarity}$												
Method	Leukemia	NSCLC	Colon	CNS	Melanoma	Ovarian	Renal	Prostate	Breast	Score	Normalized score	Normalized score$^{TRFBA\text{-}CORE}$
**CORDA**	0.02	0.01	0.02	0.02	0.01	0.01	0.01	0.04	0	0.02	0.11	0.11
**CORDA^{c}**	0.02	0.01	0.02	0.02	0.01	0.01	0.01	0.04	0	0.02	0.11	0.11
**FASTCORE**	0.05	0.14	0.06	0.08	-0.04	0.18	0.19	0.30	0.02	0.11	0.74	0.74
**FASTCORE^{c}**	0.05	0.14	0.06	0.08	-0.04	0.18	0.19	0.30	0.02	0.11	0.74	0.74
**FASTCORMICS**	0.21	0.03	0.09	0.18	-0.03	0.02	0.12	0.08	0.05	0.08	0.57	0.57
**FASTCORMICS^{c}**	0.21	0.03	0.09	0.18	-0.03	0.02	0.12	0.08	0.05	0.08	0.57	0.57
**GIMME**	0.01	0.01	0.01	0.01	0.01	0.01	0.01	0.03	0	0.01	0.07	0.07
**GIMME^{c}**	0.02	0.01	0.02	0.01	0.01	0.01	0.01	0.03	0.01	0.02	0.11	0.11
**INIT**	0.18	0.07	0.18	0.17	0.11	0.09	0.07	0.32	0.13	0.15	1	1
**INIT^{c}**	0.18	0.07	0.18	0.17	0.11	0.09	0.07	0.32	0.13	0.15	1	1
**PRIME**	NA	NA	NA	NA	NA	NA	NA	NA	NA	NA	NA	NA
**PRIME^{c}**	NA	NA	NA	NA	NA	NA	NA	NA	NA	NA	NA	NA
**TRFBA**	NA	NA	NA	NA	NA	NA	NA	NA	NA	NA	NA	NA
**TRFBA^{c}**	NA	NA	NA	NA	NA	NA	NA	NA	NA	NA	NA	NA
**iMAT**	0.04	0.02	0.04	0.04	0.03	0.03	0.03	0.08	0.03	0.04	0.26	0.26
**iMAT^{c}**	0.04	0.02	0.04	0.03	0.03	0.03	0.03	0.08	0.03	0.04	0.26	0.26
**mCADRE**	0.04	0.02	0.05	0.06	0.05	0.02	0.05	0.06	0.02	0.04	0.27	0.27
**mCADRE^{c}**	0.04	0.02	0.05	0.06	0.05	0.02	0.05	0.06	0.02	0.04	0.27	0.27
**pFBA^{c}**	NA	NA	NA	NA	NA	NA	NA	NA	NA	NA	NA	NA
**TRFBA-CORE$_{corr}$**	0.05	0.02	0.04	0.06	0.03	0.04	0.03	0.13	0.04	0.05	-	0.32
**TRFBA-CORE$^{c}_{corr}$**	0.05	0.05	0.06	0.09	0.02	0.02	0.01	0.15	0.06	0.06	-	0.38
**TRFBA-CORE$_{copt}$**	0.05	0.02	0.04	0.06	0.03	0.04	0.03	0.13	0.04	0.05	-	0.32
**TRFBA-CORE$^{c}_{copt}$**	0.05	0.05	0.06	0.09	0.02	0.02	0.01	0.15	0.06	0.06	-	0.38

8- **Performance scores for CV analyses.** Since it was not possible to directly use the results for scoring purposes, each method received a four-level score (0, 0.25, 0.75 and 1 for incapable, moderate, good and best) based on its performance in 1- recovering the missing reactions to the network ($\boldsymbol{S_1}$), and 2- robustness to the predicted growth rates ($\boldsymbol{S_2}$). The final score was calculated as the mean value of the two scores. The resulting vector of performance scores was then normalized to the maximum score (Table H).

Table H. Performance scores for cross-validation analyses. Normalized score$^{\text{TRFBA-CORE}}$ was calculated by taking into account the performance scores of TRFBA-CORE.

Method	S_1	S_2	Score	Normalized score	Normalized score$^{\text{TRFBA-CORE}}$
**CORDA**	0	0	0	0	0
**CORDA^c**	0	0	0	0	0
**FASTCORE**	0	0	0	0	0
**FASTCORE^c**	0	0	0	0	0
**FASTCORMICS**	0.75	0.75	1.50	1	1
**FASTCORMICS^c**	0.75	0.75	1.50	1	1
**GIMME**	0	0	0	0	0
**GIMME^c**	0	0	0	0	0
**INIT**	1	0	1	0.67	0.67
**INIT^c**	1	0	1	0.67	0.67
**PRIME**	0	0.75	0.75	0.50	0.50
**PRIME^c**	0	0.75	0.75	0.50	0.50
**TRFBA**	0	0.75	0.75	0.50	0.50
**TRFBA^c**	0	0.75	0.75	0.50	0.50
**iMAT**	0	1	1	0.67	0.67
**iMAT^c**	0	1	1	0.67	0.67
**mCADRE**	0	0	0	0	0
**mCADRE^c**	0	0	0	0	0
**pFBA^c**	0	0	0	0	0
**TRFBA-CORE$_{corr}$**	1	0	1	-	0.67
**TRFBA-CORE$^c_{corr}$**	1	0	1	-	0.67
**TRFBA-CORE$_{copt}$**	1	0	1	-	0.67
**TRFBA-CORE$^c_{copt}$**	1	0	1	-	0.67

9- **Performance scores for noise robustness analyses.** Similar to the CV analyses, since it was not possible to directly use the results for scoring purposes, each method received a four-level score (0, 0.25, 0.75 and 1 for incapable, moderate, good and best) based on its performance in 1- similarity levels between the generated GEMs ($\boldsymbol{S_1}$), and 2- robustness to the predicted growth rates ($\boldsymbol{S_2}$). The final score was calculated as the mean value of the two scores. The resulting vector of performance scores was then normalized to the maximum score (Table I).

Table I. Performance scores for noise robustness analyses. Normalized score$^{TRFBA\text{-}CORE}$ was calculated by taking into account the performance scores of TRFBA-CORE.

Method	S_1	S_2	Score	Normalized score	Normalized score$^{TRFBA\text{-}CORE}$
**CORDA**	0.25	0	0.25	0.13	0.13
**CORDA^c**	0.25	0	0.25	0.13	0.13
**FASTCORE**	1	0	1	0.50	0.50
**FASTCORE^c**	1	0	1	0.50	0.50
**FASTCORMICS**	1	1	2	1	1
**FASTCORMICS^c**	1	1	2	1	1
**GIMME**	0.25	0	0.25	0.13	0.13
**GIMME^c**	0.25	0	0.25	0.13	0.13
**INIT**	0.75	0	0.75	0.38	0.38
**INIT^c**	0.75	0	0.75	0.38	0.38
**PRIME**	0	0	0	0	0
**PRIME^c**	0	0	0	0	0
**TRFBA**	0	0.75	0.75	0.38	0.38
**TRFBA^c**	0	0.75	0.75	0.38	0.38
**iMAT**	0.50	1	1.50	0.75	0.75
**iMAT^c**	0.50	1	1.50	0.75	0.75
**mCADRE**	0.50	0	0.50	0.25	0.25
**mCADRE^c**	0.50	0	0.50	0.25	0.25
**pFBA^c**	0	0	0	0	0
**TRFBA-CORE$_{corr}$**	0.75	1	1.75	-	0.88
**TRFBA-CORE$^c_{corr}$**	0.75	1	1.75	-	0.88
**TRFBA-CORE$_{copt}$**	0.75	1	1.75	-	0.88
**TRFBA-CORE$^c_{copt}$**	0.75	1	1.75	-	0.88
