# Supplementary material for: A benchmark-driven approach to reconstruct metabolic networks for studying cancer metabolism
Source: PLoS Comput Biol. 2019 Apr 22;15(4):e1006936. doi: 10.1371/journal.pcbi.1006936 (PMC6497301; doi:10.1371/journal.pcbi.1006936)
Supplement: S3 Text — FASTCORMICS was assessed with regard to the assumptions made by the original implementation to improve the capability of the method to generate functional GEMs. (PDF) [file pcbi.1006936.s003.pdf]

S3 Text. **A benchmark-driven approach to reconstruct metabolic networks for studying cancer metabolism**

Oveis Jamialahmadi, Sameereh Hashemi-Najafabadi, Ehsan Motamedian, Stefano Romeo, Fatemeh Bagheri

Modification of FASTCORMICS. In our comparative analyses FASTCORMICS resulted in satisfactory performance in consistency tests for 37 out of 59 cell-specific GEMs, but among these only 15 of them were capable of predicting a non-zero growth rate. FASTCORMICS was developed based upon FASTCORE, which provides the possibility of defining non-penalized reactions in addition to core and non-core reactions [1]. In the original FASTCORE implementation, L1-norm of non-core reaction fluxes (P) is minimized while core reactions (K) are forced to carry a minimum flux value. In this case, some core reactions may be removed incorrectly as the result of L1-norm minimization. To tackle this issue, the scaled form of ε ($\times 10^5$) was employed to find sparse modes [2]. Nevertheless, the evaluation of the scaling factor showed that the ability of the algorithm to include core reactions in the final GEM, is highly dependent on this scaling factor. This is due to the fact that the *FindSparseMode* function of FASTCORE forces the core reactions set (K) to carry flux values, which may lead to an infeasible solution. Reducing the scaling factor enables the algorithm to force more core reactions to carry a positive flux value; however, as mentioned above, scaling factor cannot be lowered below a certain threshold. Sensitivity analysis of scaling factor revealed that the optimal value for scaling factor is 10^3 (Figure A).

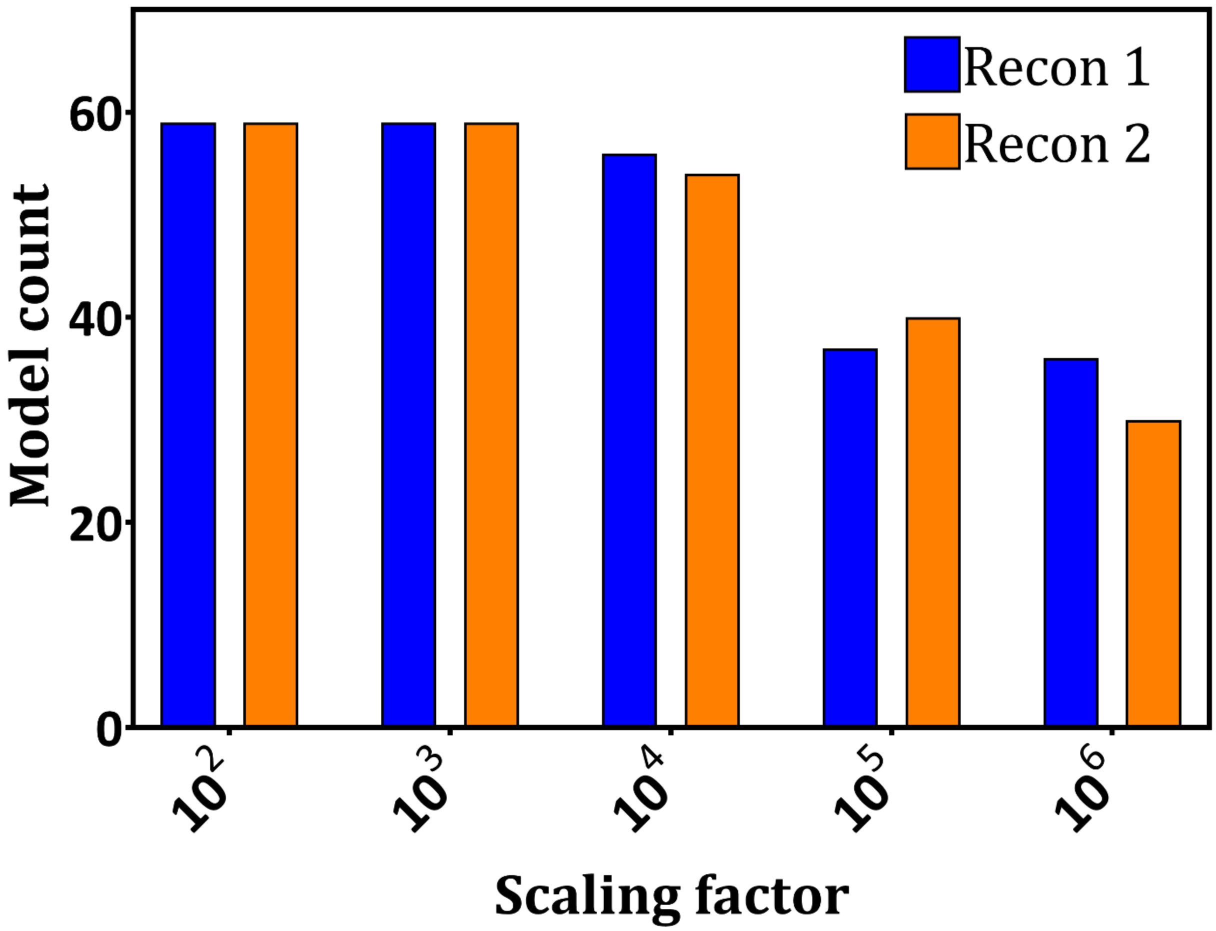

Figure A. Sensitivity analysis of scaling factor. Different scaling factors were examined to evaluate the effect of this parameter on the capability of FASTCORMICS to generate non-empty GEMs.

Furthermore, FASTCORMICS considers reactions corresponding to z-scores below 0 as inactive, and sets their upper and lower bounds to zero [1]. However, we observed that this step resulted in the elimination of the biomass reaction from the final GEM for several cell lines, which explains the low number of models with the ability of predicting a non-zero growth rate (Figure B).

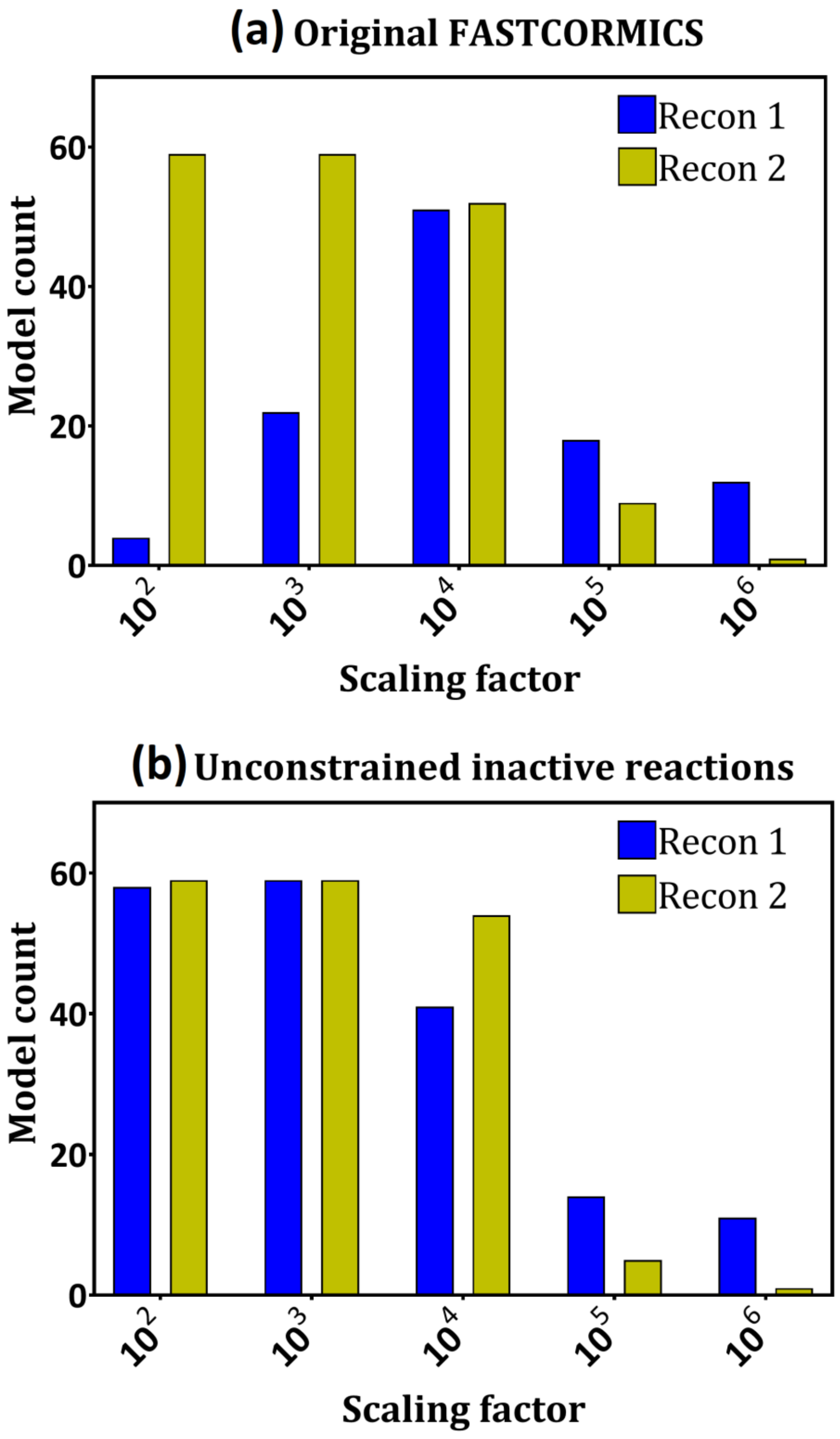

Figure B. The effect of inactive reaction constraints. The ability of FASTCORMICS to generate GEMs capable of predicting non-zero growth rates was evaluated for (a) original implementation of FASTCORMICS, and (b) FASTCORMICS with unconstrained inactive reactions (z-score < 0).

We observed that FASTCORMICS with the following modifications performs better than the original one: 1) scaling factor of 10^3 (Figure A), and 2) removing inactive reaction constraining step (Figure B). Therefore, we referred to these modifications as “modified FASTCORMICS” within the main text.

References

1. Pacheco MP, John E, Kaoma T, Heinäniemi M, Nicot N, Vallar L, et al. Integrated metabolic modelling reveals cell-type specific epigenetic control points of the macrophage metabolic network. BMC Genomics. 2015; 16:809.
2. Vlassis N, Pacheco MP, Sauter T. Fast reconstruction of compact context-specific metabolic network models. PLoS Comput Biol. 2014; 10(1):e1003424.
