# Supplementary figures and images for: A benchmark-driven approach to reconstruct metabolic networks for studying cancer metabolism
Source: PLoS Comput Biol. 2019 Apr 22;15(4):e1006936. doi: 10.1371/journal.pcbi.1006936 (PMC6497301; doi:10.1371/journal.pcbi.1006936)

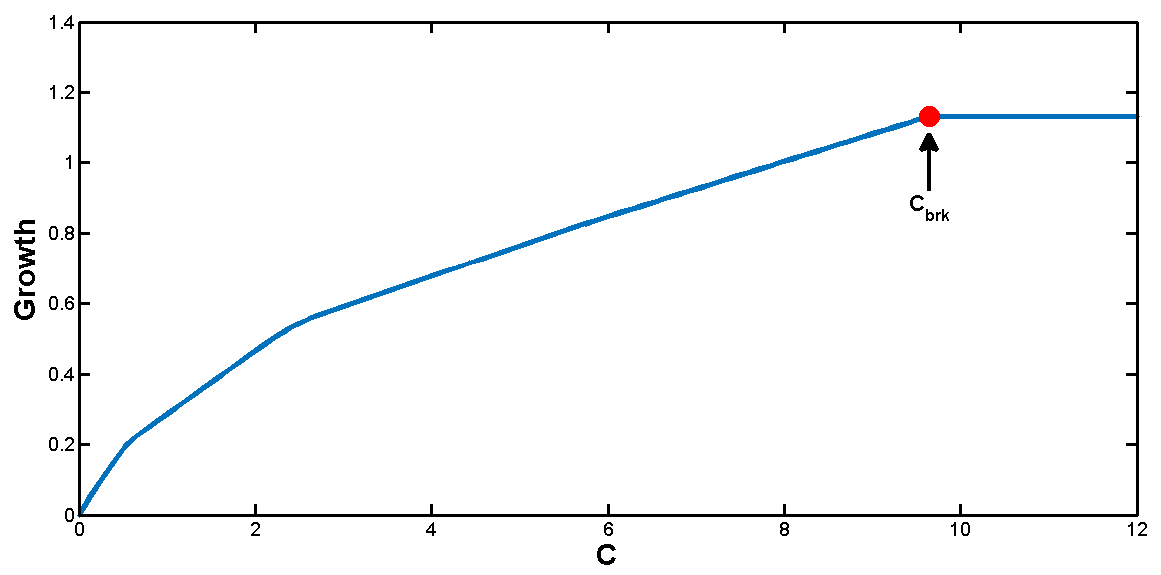

Supplement: S1 Fig — The breaking point (Cbrk) denotes the C value at which further reduction in C affects the predicted growth rate. (TIF) [file pcbi.1006936.s004.tif]

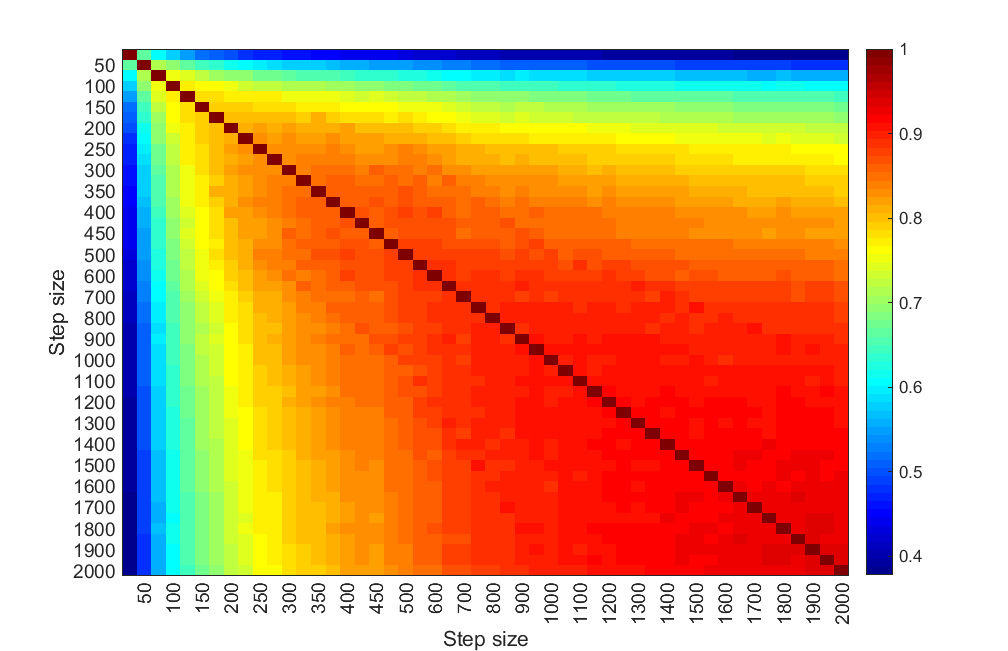

Supplement: S2 Fig — The indices are shown as the mean value across all cell lines in NCI-60 panel. (TIF) [file pcbi.1006936.s005.tif]

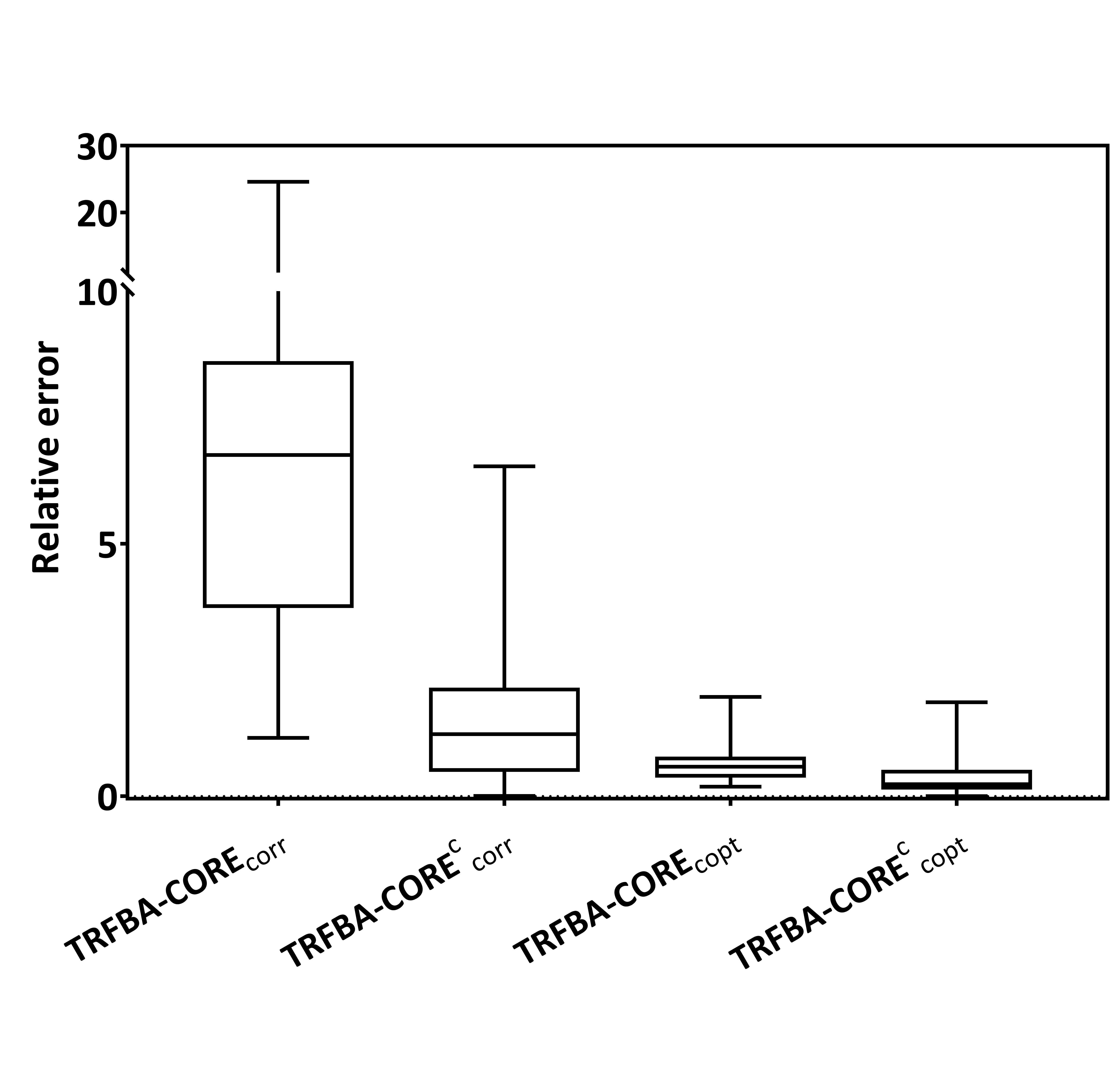

Supplement: S3 Fig — Distribution of relative error for prediction of growth rates for 4 variations of TRFBA-CORE (with general/cell-specific media, and Copt/Ccorr). Each box-plot shows the distribution of error across all cell lines in NCI-60 panel. (TIF) [file pcbi.1006936.s006.tif]

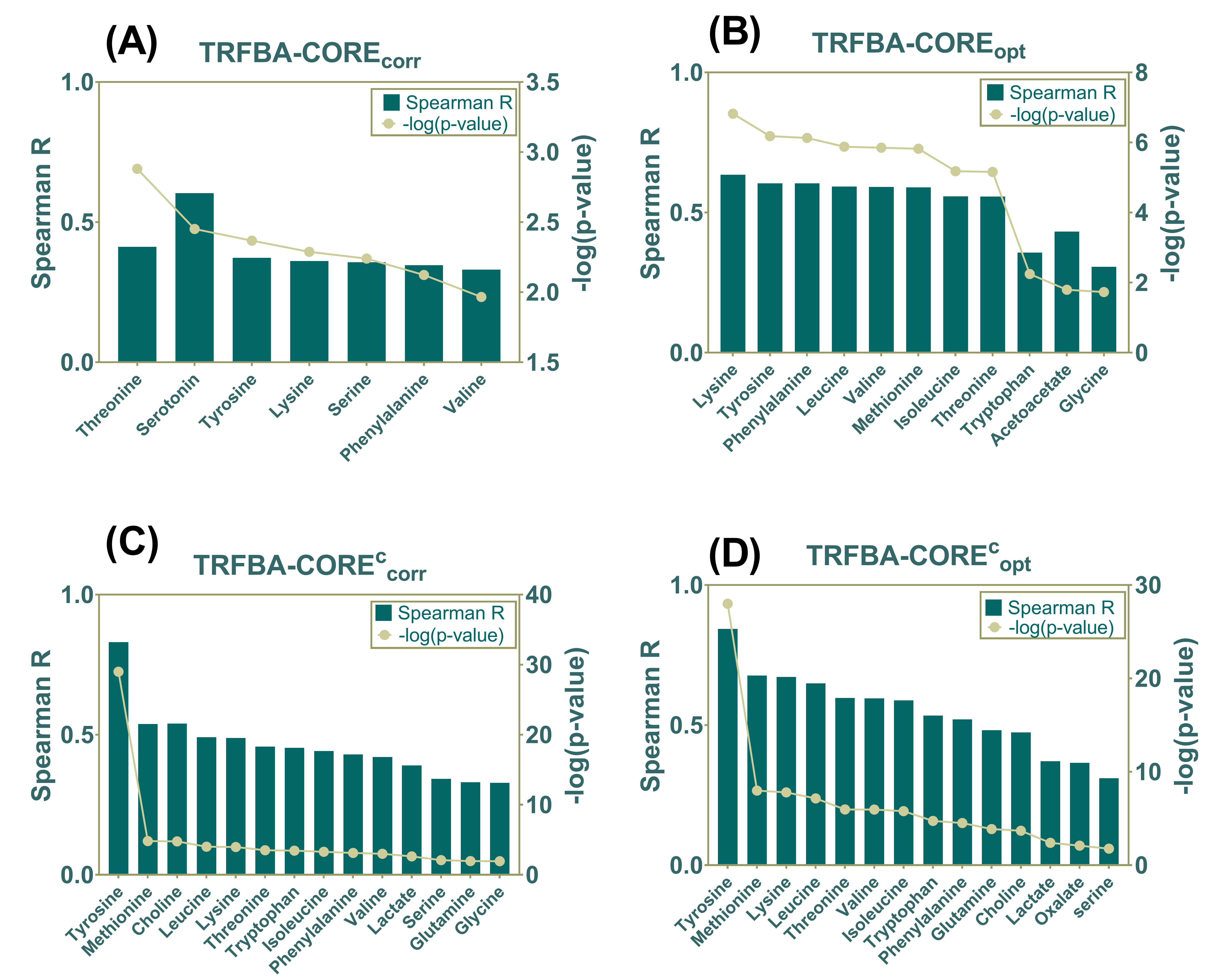

Supplement: S4 Fig — Spearman correlation between measured and predicted uptake/secretion flux rates of metabolites for (A) TRFBA-COREcorr, (B) TRFBA-COREopt, (C) TRFBA-COREccorr, and (D) TRFBA-COREcopt. Represented p-values were adjusted for False discovery rate (α = 0.05). (TIF) [file pcbi.1006936.s007.tif]

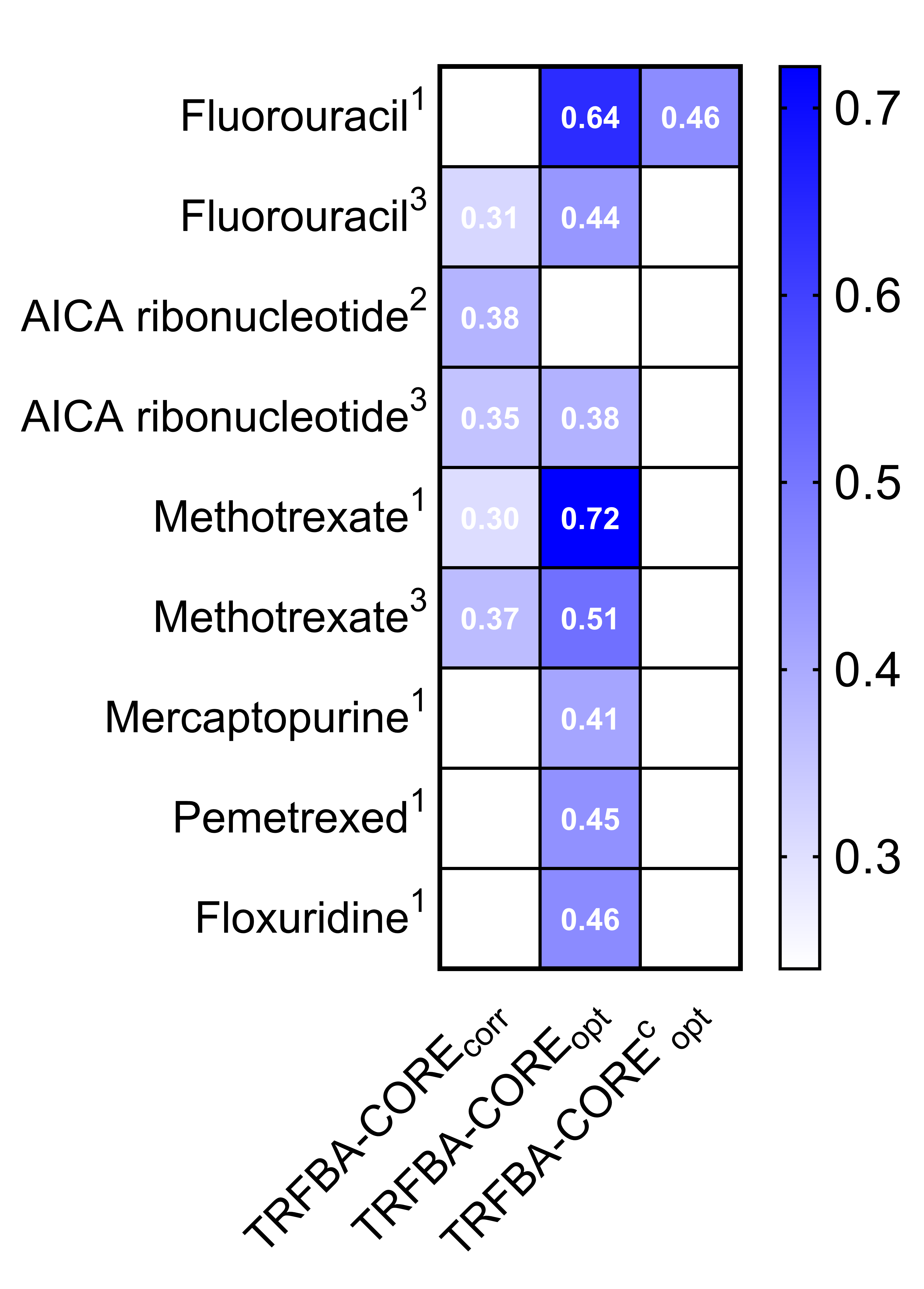

Supplement: S5 Fig — Heatmap of significant Spearman correlations between simulated and experimental drug response data for 4 variations of TRFBA-CORE (with general/cell-specific media, and Copt/Ccorr). The Spearman coefficients for each drug have been shown on the figure. Superscripts indicate drug response data taken from (1) Holbeck et al [38], (2) Garnett et al [39] and (3) Yang et al [40]. (TIF) [file pcbi.1006936.s008.tif]

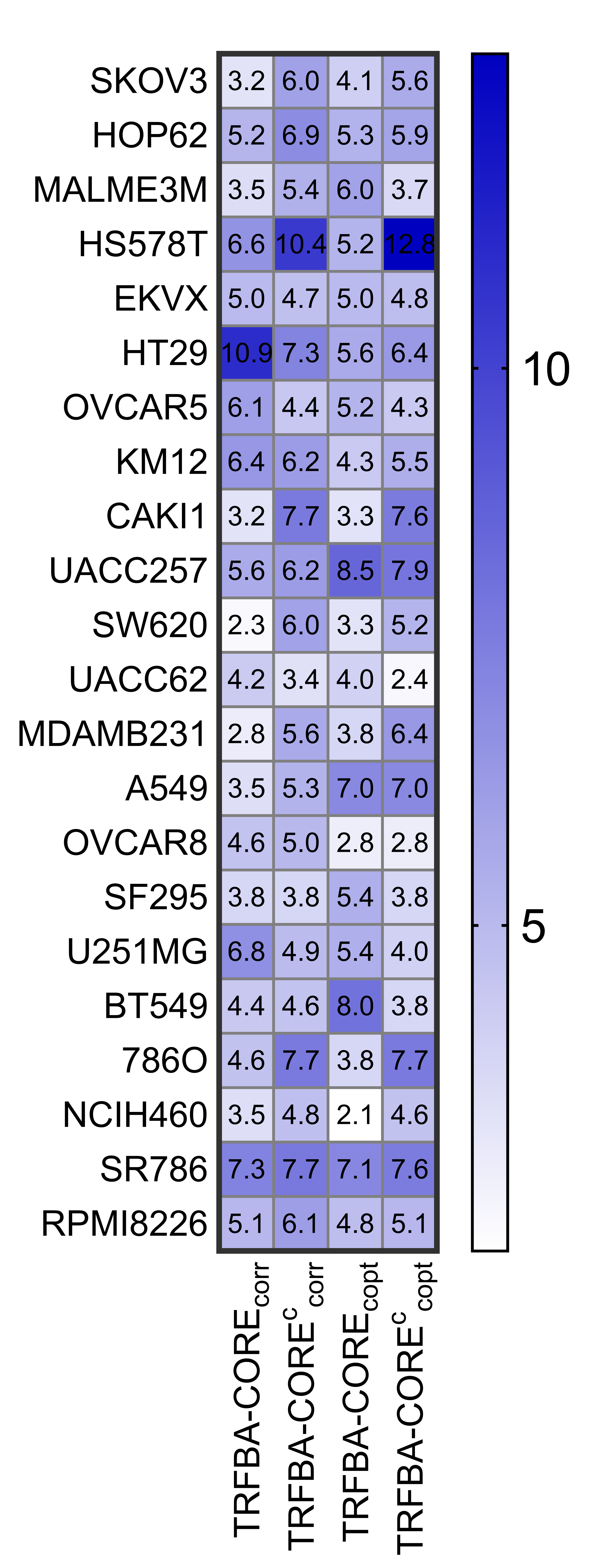

Supplement: S6 Fig — Heatmap of enrichment p-values for predicted cell-line specific essential genes for 4 variations of TRFBA-CORE (with general/cell-specific media, and Copt/Ccorr). The numbers indicate -log10 enrichment p-values. (TIF) [file pcbi.1006936.s009.tif]

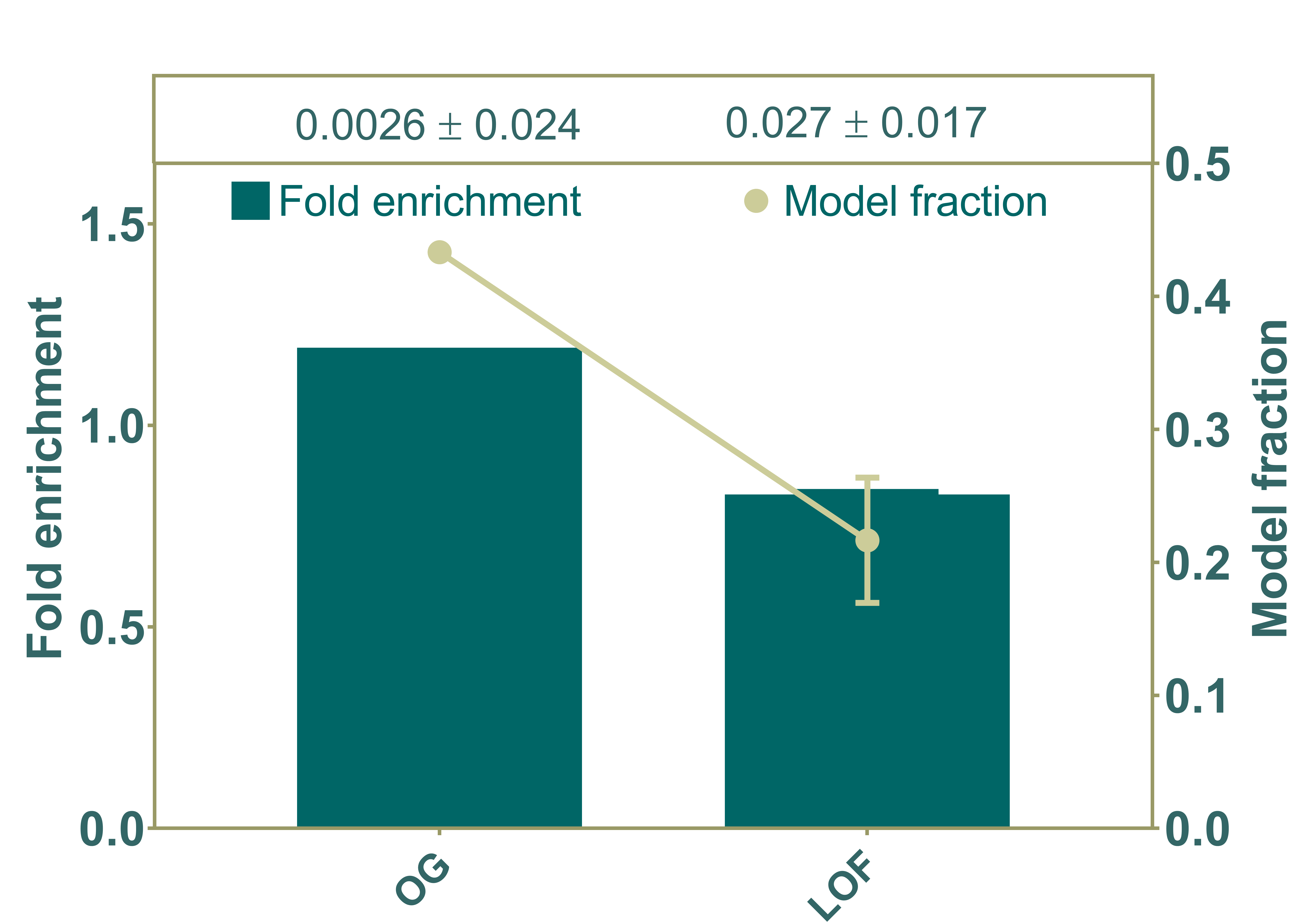

Supplement: S7 Fig — Mean enrichment of predicted OGs and and LOFs with experimental data. The error bar indicates the standard deviation across GEMs generated with general and cell-specific media. Hypergeometric p-values are shown above the figure. Model fraction represents the fraction of generated GEMs with significant p-values (<0.05). (TIF) [file pcbi.1006936.s010.tif]

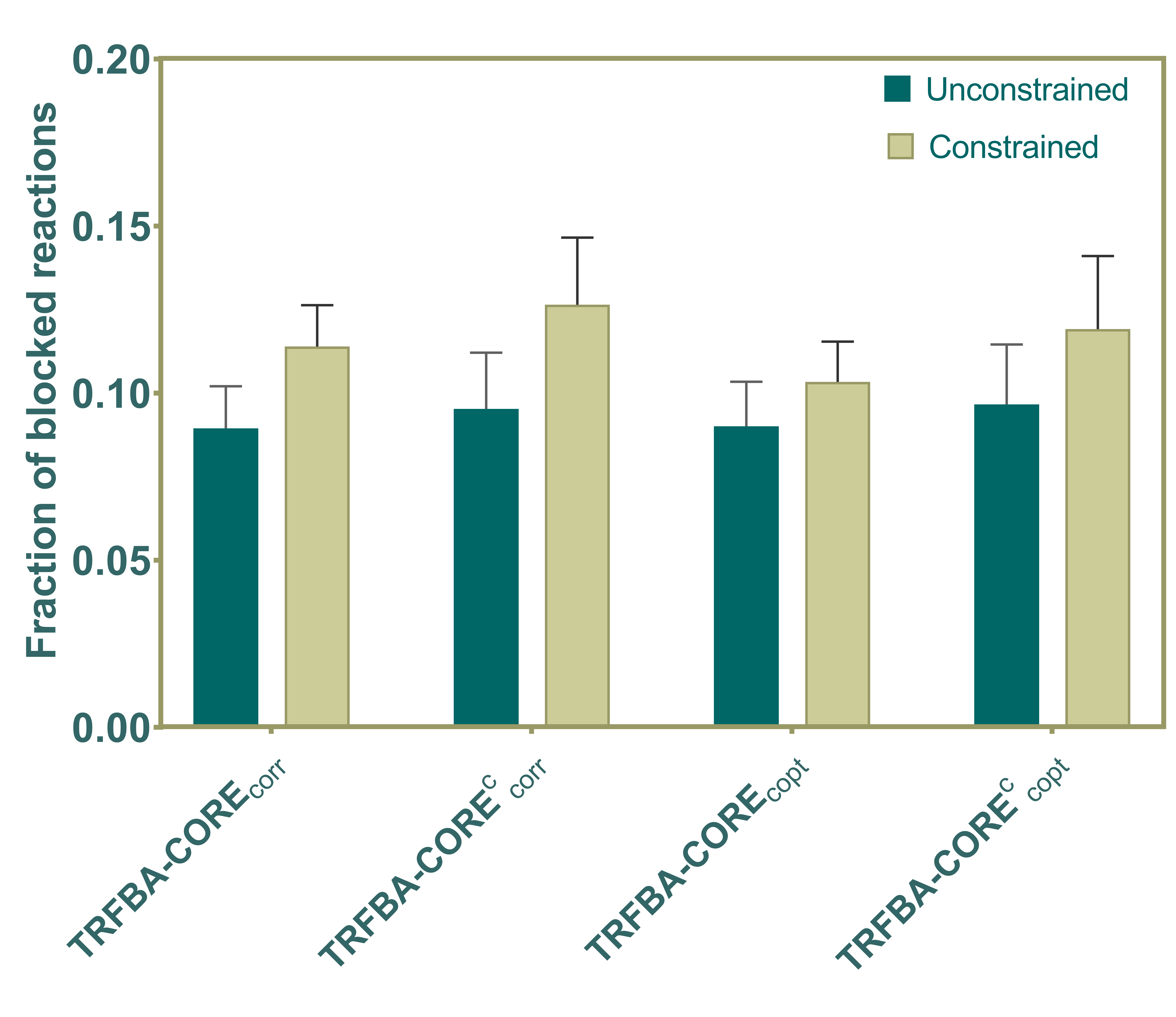

Supplement: S8 Fig — The presence of blocked reactions were assessed in both constrained and unconstrained states for 4 variations of TRFBA-CORE (with general/cell-specific media, and Copt/Ccorr). Data shown as mean fraction of existing blocked reaction across all generated GEMs, and error bars represent the standard deviation. (TIF) [file pcbi.1006936.s011.tif]

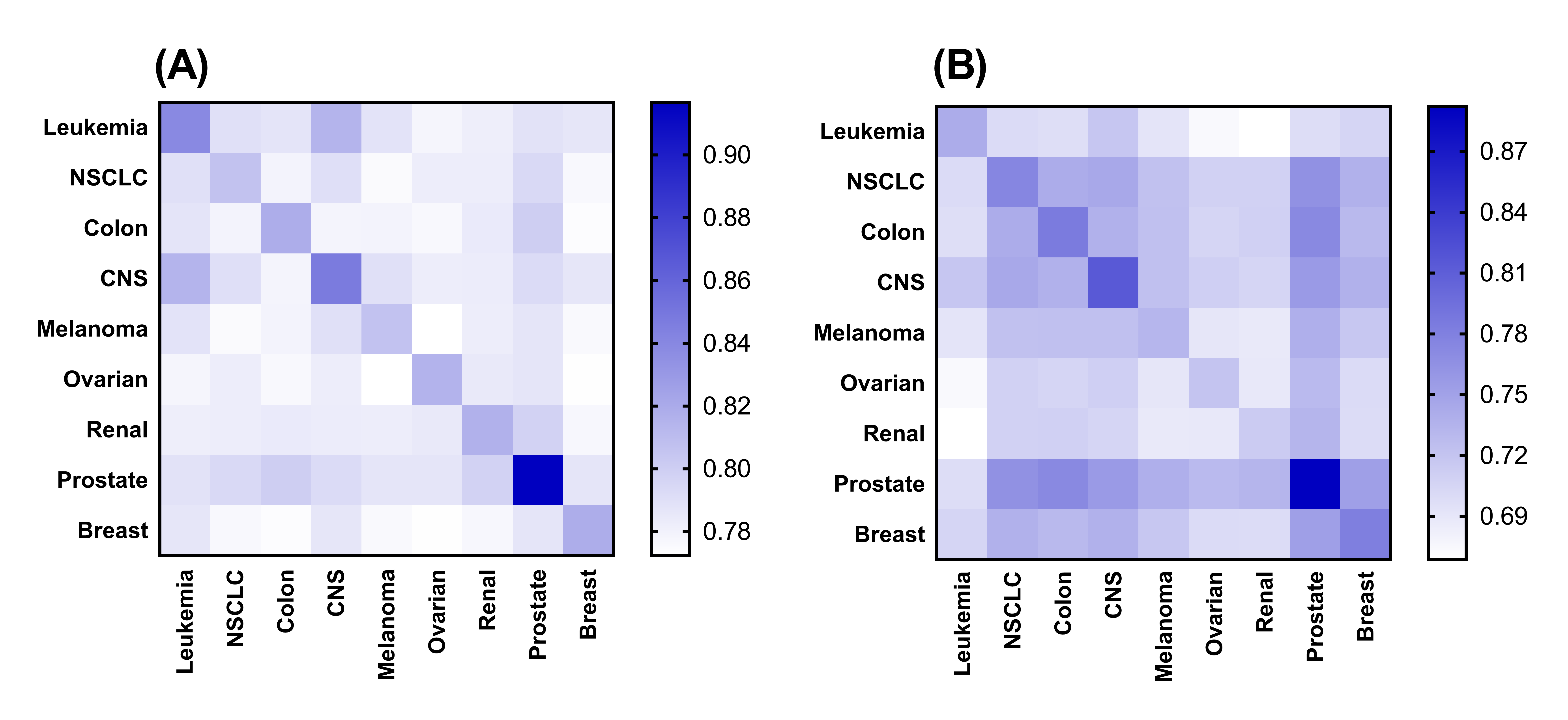

Supplement: S9 Fig — Average Jaccard similarity index computed for GEMs built by (A) TRFBA-CORE and (B) TRFBA-COREc. Each square represents the average pairwise Jaccard value for each cancer type in the NCI-60 panel. (TIF) [file pcbi.1006936.s012.tif]

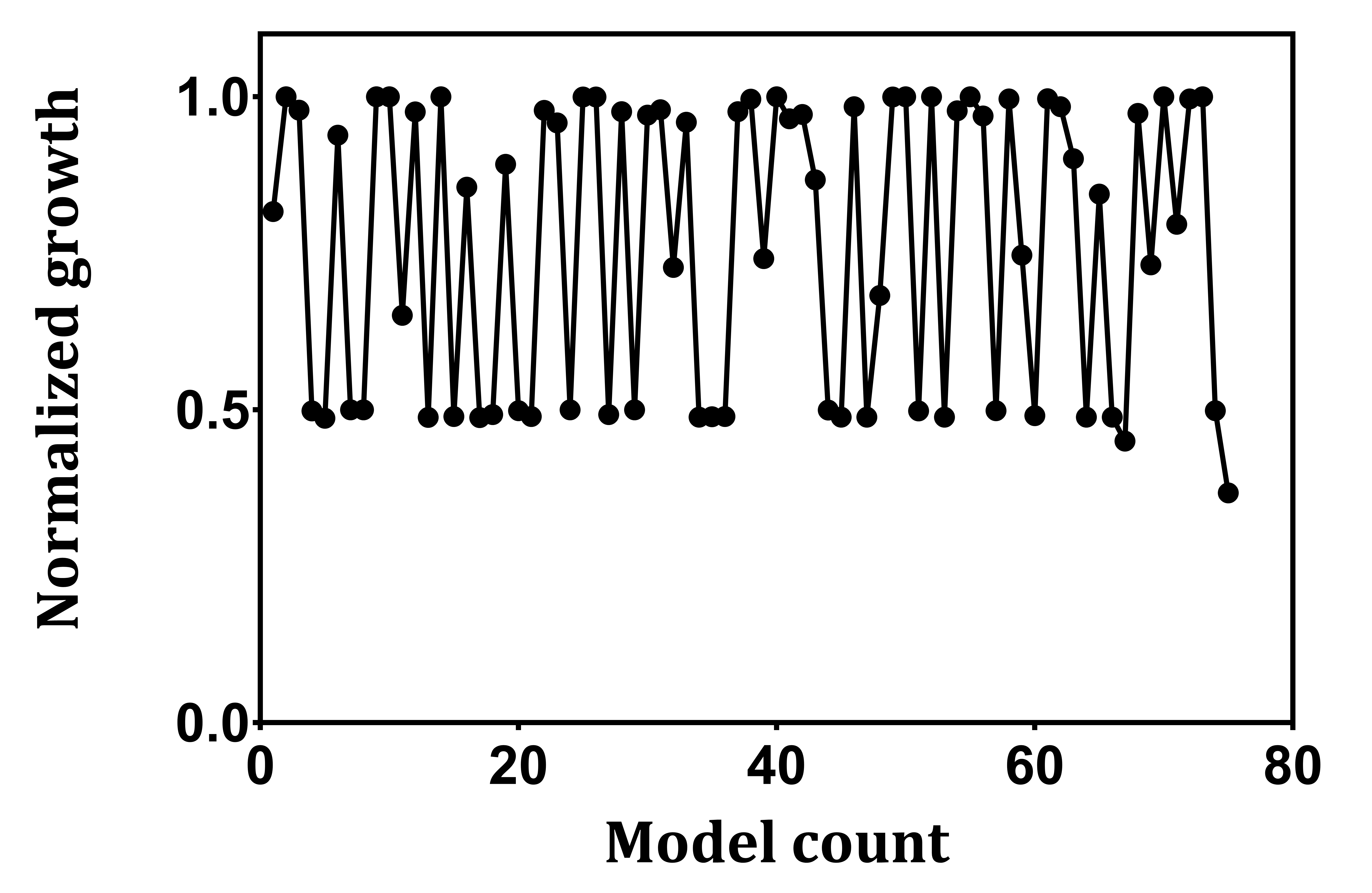

Supplement: S10 Fig — Model count represents the GEMs generated by incomplete growth-correlated reactions in the input. (TIF) [file pcbi.1006936.s013.tif]

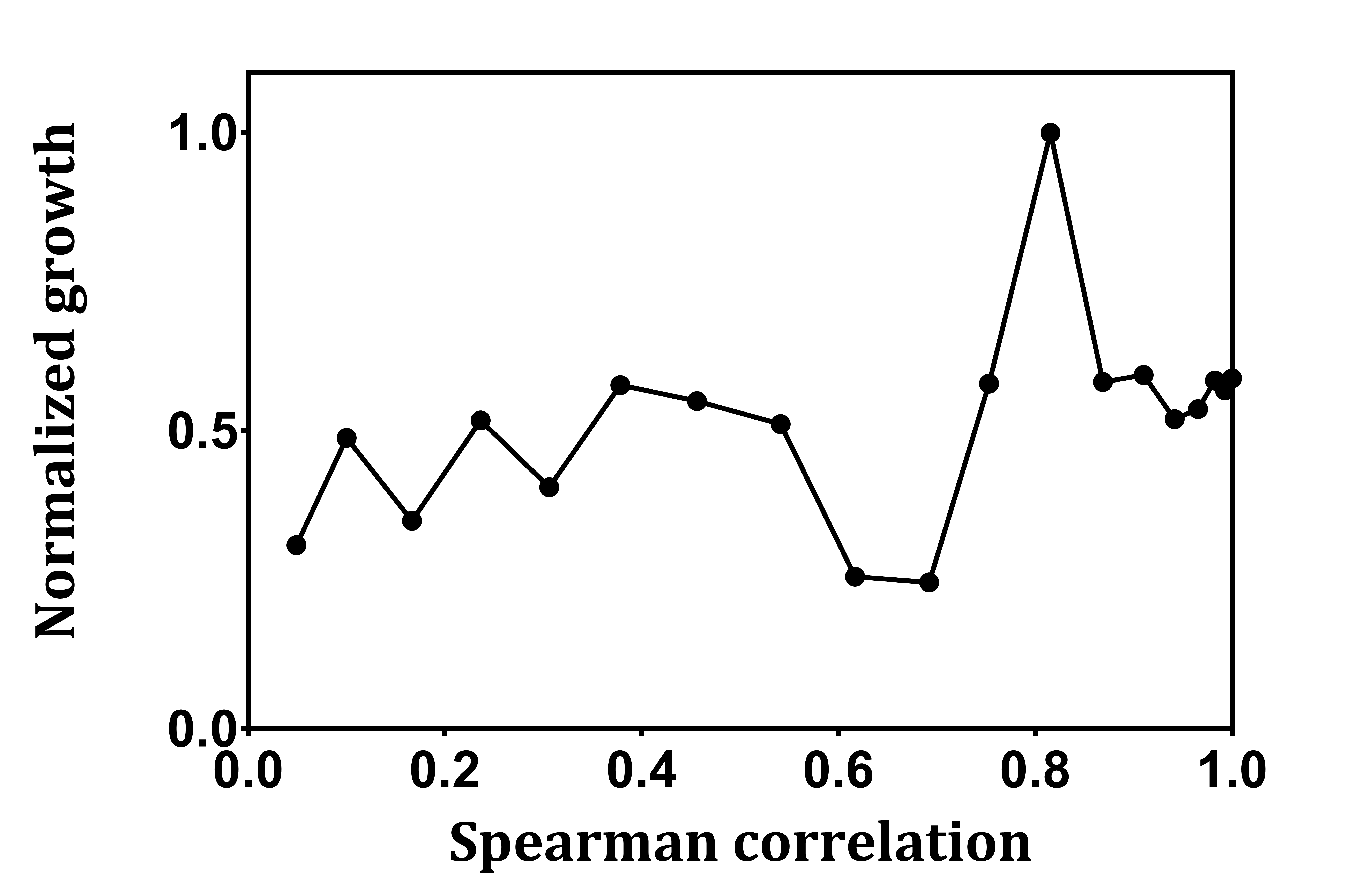

Supplement: S11 Fig — The x-axis shows the spearman correlation coefficient between each set of noisy data and original expression profile ranging from 1 (original) to R < 0.004 (random). (TIF) [file pcbi.1006936.s014.tif]

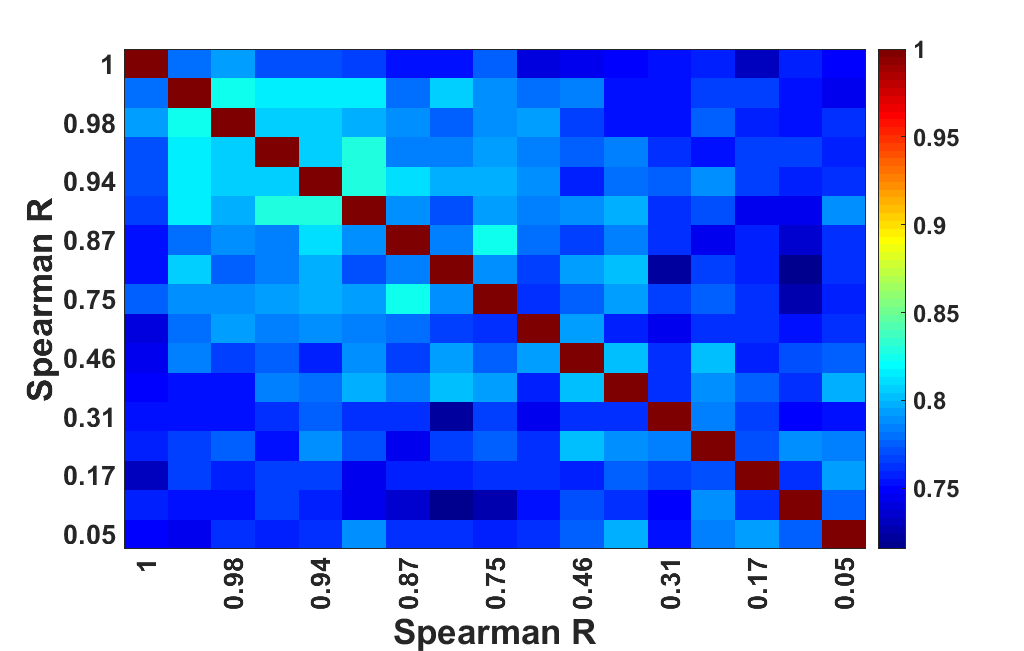

Supplement: S12 Fig — (TIF) [file pcbi.1006936.s015.tif]
